# A chromosome-level genome reveals genome evolution and molecular basis of anthraquinone biosynthesis in *Rheum palmatum*

**DOI:** 10.1186/s12870-024-04972-2

**Published:** 2024-04-10

**Authors:** Tianyi Zhang, Lipan Zhou, Yang Pu, Yadi Tang, Jie Liu, Li Yang, Tao Zhou, Li Feng, Xumei Wang

**Affiliations:** School of Pharmacy, Xi’an Jiaotong University, Xi’an, 710061 China

**Keywords:** *Rheum palmatum*, Genome, Transposable element, Anthraquinone, Whole genome duplication

## Abstract

**Background:**

Rhubarb is one of common traditional Chinese medicine with a diverse array of therapeutic efficacies. Despite its widespread use, molecular research into rhubarb remains limited, constraining our comprehension of the geoherbalism.

**Results:**

We assembled the genome of *Rheum palmatum* L., one of the source plants of rhubarb, to elucidate its genome evolution and unpack the biosynthetic pathways of its bioactive compounds using a combination of PacBio HiFi, Oxford Nanopore, Illumina, and Hi-C scaffolding approaches. Around 2.8 Gb genome was obtained after assembly with more than 99.9% sequences anchored to 11 pseudochromosomes (scaffold N50 = 259.19 Mb). Transposable elements (TE) with a continuous expansion of long terminal repeat retrotransposons (LTRs) is predominant in genome size, contributing to the genome expansion of *R. palmatum*. Totally 30,480 genes were predicted to be protein-coding genes with 473 significantly expanded gene families enriched in diverse pathways associated with high-altitude adaptation for this species. Two successive rounds of whole genome duplication event (WGD) shared by *Fagopyrum tataricum* and *R. palmatum* were confirmed. We also identified 54 genes involved in anthraquinone biosynthesis and other 97 genes entangled in flavonoid biosynthesis. Notably, *RpALS* emerged as a compelling candidate gene for the octaketide biosynthesis after the key residual screening.

**Conclusion:**

Overall, our findings offer not only an enhanced understanding of this remarkable medicinal plant but also pave the way for future innovations in its genetic breeding, molecular design, and functional genomic studies.

**Supplementary Information:**

The online version contains supplementary material available at 10.1186/s12870-024-04972-2.

## Background

Polygonaceae is a widespread plant family, comprising approximately 1200 species divided into 46–50 genera [1–4]. Many species within this family hold substantial values in human activities, including crops and vegetables, i.e. buckwheat, *Coccoloba uvifera*, *Rumex acetosa* and *Rheum rhabarbarum*, and ornamentals, i.e. *Antigonon leptopus* and *Persicaria perfoliata*. Additionally, Polygonaceae species also have medicinal properties, with examples including *Pleuropterus multiflorus*, *Reynoutria japonica*, and rhubarb.

Rhubarb’s medicinal use dates back to ancient China, with its first documentation in “*Shen Nong Ben Cao Jing*”. It was also mentioned under the name “ῥᾶ” (ra in Latin) in *De Materia Medica* [5], indicating a long history of medicinal use in the old world. Modern pharmacological studies have indicated rhubarb’s effectiveness as a purgative, detoxification, blood stasis removal, diuresis, and antibacterial medicine. According to the *Chinese Pharmacopoeia* (Edition 2020) [6], *Rheum palmatum* L., *Rheum tanguticum* (Maxim. ex Regel) Maxim. ex Balf., and *Rheum officinale* Baill. are three source plants of rhubarb. While some relatives, such as *Rumex* spp., *Re. japonica*, *R. rhabarbarum*, and *Rheum nobile*, are sometimes used as counterfeits of rhubarb likewise [7]. Morphology and molecular evidence suggests that the three source plants of rhubarb should be treated as a species complex, namely *Rheum palmatum* complex (RPC) [7–12]. RPC contains over one hundred compounds, including anthraquinone, bianthrone, corresponding glycoside, flavonoids, phenolic acids, chromone, and butyrophenone [13, 14]. Notably, total anthraquinone and free anthraquinone are considered as the qualification indicators of rhubarb due to their potency in affecting the digestive tracts and other organs. Flavonoids and phenolic acids are also thought to contribute to rhubarb’s efficacy.

Empirical studies have uncovered four critical pathways for anthraquinone accumulation in plants, including shikimate, mevalonate (MVA), methyl erythritol phosphate (MEP) and polyketide pathways [15]. The shikimate pathway is shared with the biosynthesis of indole alkaloids, and the MVA and MEP pathways provide common precursors for terpene and alizarin-type anthraquinone biosynthesis. On the contrary, the polyketide pathway is primarily responsible for emodin-type anthraquinone biosynthesis, albeit with several unclear reactions. It is speculated that a polyketide synthase (PKS) enzyme condenses seven malonyl-CoA and one acetyl-CoA into octaketide which then undergoes dehydration, enolization and oxidation to form emodin and chrysophanol, two basic anthraquinones. In *Aloe arborescens*, AaOKS has been found to facilitate the formation of SEK4 and SEK4b aromatic octaketides [16, 17]. Similarly, RjOKS has been confirmed as the key enzyme of polyketide pathway in *Re. japonica* [18]. In *R. palmatum*, four PKS enzymes—RpALS, RpBAS and two RpCHSs—have been identified, but none can catalyze the production of polyketide compounds [19–21]. It is worth noting that a reductase (polyketide reductase, PKR) and a cyclase (polyketide cyclase, PKC) are speculated to catalyze subsequent process following octaketide formation [22, 23]. Further modifications like oxidation, methylation and glycosylation are believed to be catalyzed by cytochrome P450 monooxygenases (CYP), *O*-methyl transferases (OMT) and UDP-glucuronosyltransferase (UGT), leading to the formation of various anthraquinone and the glycosides. One UGT protein, RpUGT1, has been identified as responsible for converting emodin to emodin-6-O-glucoside [24]. However, little is known about other modifications in rhubarb, such as oxidation and methylation [25].

The unraveling of hundreds of medicinal plant genomes has been deepened our understanding of the genetic underpinnings behind the biosynthesis of medicinally active compounds in herbal species. For instance, a CHS-L potentially involved in anthraquinone biosynthesis has been identified in *Senna tora* [26], while the expansion of CYP725A sheds light on paclitaxel accumulation in *Taxus* [27, 28]. Moreover, significant genomic differences have been observed not only among different species’ genomes but also within the same species, primarily as a result of environmental adaptation. Evolutionary processes, including transposable element (TE) dynamics, genome duplication, and chromosome rearrangement, have impacted the genome characters, leading to structural variations, gene birth and death, and co-expression of gene clusters that aid in responding to environmental pressures. For instance, the efficient elimination of TEs has reduced the genome size in *Utricularia gibba* and *Arabidopsis thaliana*, indicating an alternative strategy for proliferation and survival with decreased genome copying costs [29]. Whole genome duplication provides the raw materials for neofunctionalization due to functional redundancy, which could benefit fitness enhancement and niche expansion in *Trifolium repens* [30]. Moreover, a recent study has demonstrated that several genes were recruited into the benzylisoquinoline alkaloid (BIA) gene cluster by chromosome fusion, translocation and duplication in *Papaver somniferum*, resulting in higher morphine and noscapine accumulation compared to *Papaver setigerum* and *Papaver rhoeas* [31].

In Polygonaceae, at least fifteen whole genome sequencing studies have been conducted, with five focusing on the genus *Rheum*, i.e. *Rheum alexandrae* [32], *R. nobile* [33, 34], and two recently published genomes, *R. tanguticum* [35] and *R. officinale* [36], uncovering large genome sizes of 2.76 Gb and 7.68 Gb, respectively. Analyses of these genomes have identified that three chalcone synthases (CHSs), four CYP, and two β-glucosidases (BGLs) with strong correlations to anthraquinone accumulation in *R. tanguticum*, alongside 666 candidate genes potentially involved in anthraquinone biosynthesis within the genome of *R. officinale*. Despite this progress, our understanding of genome dynamics in evolution and the identification of genes related to secondary metabolism remains elusive.

RNA sequencing studies in RPC are shedding light on the intricacies of anthraquinone biosynthesis in rhubarb. Our previous studies had pinpointed candidate unigenes engaged in the MVA, MEP, shikimate and polyketide pathways in RPC [37, 38], providing valuable insights into the molecular basis of the organ-specific gene expression in rhubarb. However, the genetic mechanism of the anthraquinone biosynthetic pathway in RPC is still largely unknown. To address this knowledge gap, this study conducted comprehensive genome sequencing of RPC using a combination of HiFi, ONT, Illumina, and Hi-C techniques. A chromosome-level rhubarb genome was assembled with high quality (scaffold N50 = 259.19 Mb). Our aim is to offer a comprehensive understanding of genome characteristics and dynamics within Polygonaceae (buckwheat family) and identify candidate genes involved in biosynthesis pathways for both anthraquinones and flavonoids in rhubarb. Ultimately, these findings are expected to benefit molecular function elucidation, plant breeding, and conservation efforts for RPC.

## Methods

### Plant materials

Fresh leaves were collected from an individual of *R. palmatum* that was being cultivated in greenhouse conditions on the campus of Xi’an Jiaotong University (34°13’N, 108°56’E) for whole genome sequencing. Approximately 3 g of young leaf tissue was cleaned and stored in liquid nitrogen before DNA extraction. In addition, we also collected three wild accessions each with different organs (root, R; stem, S; leaf, L; flower, F) from two distinct populations QHZK (35°18’N, 101°56’E) and SNPL (32°01’N, 109°21’E). These samples were then stored at -80 °C before sequencing.

### Library construction and sequencing

An optimized cetyl trimethyl ammonium bromide (CTAB) method was adopted for DNA extraction under the guide of the protocol [39]. The total RNA from the aforementioned 24 samples was extracted separately following the manual of the RNeasy Plant Mini Kit (Qiagen, Valencia, CA). The quality and quantity of the extracted DNA were assessed by 1.2% gel electrophoresis analysis (Life Technologies, CA) and NanoDrop 2000 analysis (Thermo Fisher Scientific, USA). On the other hand, RNA integrity and quantity were determined using 2% gel electrophoresis experiment in combination with NanoDrop 2000 analysis.

The qualified DNA underwent random fragmentation mediated by Megaruptor (Diagenode, Belgium), followed by a repair process to eliminate damaged bases. Subsequently, adaptors were ligated to construct a 20 kb library suitable for HiFi sequencing, employing SMRTbell® Express Template Preparation Kit (PacBio, USA) in accordance with the manufacturers’ instruction. Meanwhile, DNA fragments approximating 15 kb in length underwent size selection via Pippin HT (Sage science, USA) and were ligated to adaptors using the 1D ligation sequencing kit (ONT, Oxford, UK). These fragments then underwent gap repair to facilitate the construction of a library for ONT sequencing. A paired-end 150 bp library was also built following the protocol of the Illumina HiSeq platform (Illumina, USA) for genome size estimation. Whole genome sequencing for our focal species was conducted on three different platforms, including MGISEQ2000 (BGI, China), Sequel II (PacBio, USA) and Nanopore (Oxford, UK). Raw reads were filtered with sequencing quality > Q30 using FastQC v0.11.9 (https://www.bioinformatics.babraham.ac.uk/projects/fastqc/). This process involved the removal of adaptors and duplicated reads. Besides, we constructed a Hi-C sequencing library using small pieces of fresh leaves, which was lysed, biotin-labeled, purified, fragmented and treated by 2% formaldehyde solution prior to constructing a pair-end 150 bp library on the MGISEQ2000 platform (BGI, China). The RNA library was constructed following the manufacturer’s protocols with high integrity RNA and sequenced on the Illumina HiSeqX platform to obtain raw paired-end 150 bp reads. FASTQC was used to remove adapters and low-quality reads to obtain clean reads. The total number of clean reads varied between samples, ranging from 23,931,550 (flower5, QHZK) to 39,364,810 (leaf4, QHZK).

### Genome size estimation, assembly and quality assessment

Genome size, heterozygosity, and repeat content estimations were performed using the *k*-mer frequency method [40]. Initially, fastp v0.19.4 was employed to filter short reads with default parameters [41]. Subsequently, *k*-mer frequencies (*k*-mer size = 19) were counted utilizing Jellyfish v2.3.0 [42]. Finally, the output file from Jellyfish as an input of GenomeScope v2.0 [43]. Based on these evaluations, the genome size of *R. palmatum* was estimated to be ∼ 2.8 Gb (Figure S1).

Hifiasm v0.14-r312 was utilized for assembling the long and highly-accuracy HiFi reads with default parameters [44]. The resulting sequence graphs were converted from GFA to FASTA format via Gfatools v0.5-r234 [45]. Prior to de novo assembly of the *R. palmatum* genome using wtdbg2 v2.3 with default settings [46], we used NextDenovo v2.5.0 (https://github.com/Nextomics/NextDenovo) for self-correction of the ONT long reads. After conducting a comparison of the quality between the raw contigs assembled from HiFi and ONT long reads, it was observed that the former demonstrated superior quality. Consequently, it was selected for the subsequent assembly process, while the ONT reads were employed for the correction and validation of the contigs. The obtained contigs were polished using NextPolish v1.01 [47], involving three rounds of alignment with both long and short reads. The high-quality Hi-C data were mapped to these contigs using Burrows-Wheeler Aligner (BWA) v0.7.17 [48], and the uniquely mapped reads were selected for further analysis. 3D-DNA v180922 (https://github.com/aidenlab/3d-dna) was utilized to anchor the contigs into pseudochromosomes [49]. Juicebox v1.9.9 (https://github.com/aidenlab/Juicebox) was then applied for visualizing chromatin interactions and manual corrections (Figure S2) [50].

To ensure the quality of our genome assembly, we implemented four evaluation methods: (i) mapping short reads to our final assembly via BWA to calculate the overall mapping rates and coverage; (ii) assessing the completeness of the final assembly with BUSCO (Benchmarking Universal Single-Copy Orthologs) v5.5.0 with the embryophyta_odb10 database [51]; (iii) estimating the long terminal repeat (LTR) assembly index (LAI) via LTR_retriever v2.9.0, where an LAI > 20 indicates a high-quality genome benchmarking [52]; and (iv) determining the consensus quality value (QV) score by Mercury, where a high QV score implies accurate genome consensus [53]. Additionally, the circos v0.69.9 (http://circos.ca/) was used for visualizing gene density, GC content, repeat content, and gene synteny on the individual pseudochromosomes of *R. palmatum.*

### Genome annotation

We integrated *de novo* and homology-based methods to identify repeat sequences in the genome of *R. palmatum*. Firstly, we utilized RepeatMasker v4.1.0 (https://www.repeatmasker.org/) to identify homologous repeat sequences by referencing the Repbase v20181026 (https://www.girinst.org/repbase/) and Dfam v3.5 (https://dfam.org/release/) database. Secondly, we employed LTR_finder v1.0.7 [54], LTR_harvest v1.6.2 [55], and RepeatModeler v1.0.8 to search the repeat sequences in *R. palmatum* genome, which aided in constructing a *de novo* repeat library. All identified repeat sequences were then combined and served as an integrated library for RepeatMasker. Additionally, we also detected tandem repeats using tandem Repeat Finder (TRF) v.4.09.1 [56] with parameter “1 1 2 80 5 200 2000 -d” [57].

For identifying LTRs, we used LTR_finder and LTR_harvest, and then integrated the obtained LTRs via LTR_retriever. MUSCLE v3.8.31 (https://drive5.com/muscle/) was employed for aligning the flanking sequences of intact LTR-RTs. We estimated the insertion times of LTR-RTs using a formula T = K/2r based on genetic distance and neutral mutation rate, where K can be estimated with the formula K = -0.75 × ln(1–4λ/3) [58], the parameter r represents neutral mutation rate and can be set as 7.0 × 10^− 9^ substitutions/site/year in line with previous studies [59, 60]. TEsorter v1.4.6 was used to classify intact LTRs into family-level categories with default parameters [61]. Information of full-length LTRs for related species of *R. palmatum* was downloaded from MBKBase (http://mbkbase.org/Pinku1/) or the NCBI genome database (Table S1). We inferred the phylogeny of Gypsy and Copia subclades based on aligned reverse transcriptase (RT) sequences using fasttree v2.1.1 with parameters “-spr 4 -gamma -fastest -no2nd -pseudo -boot 1000” [62] and visualized the result utilizing the R package *ggtree* v3.7.5 [63].

We also attempted to discover telomere and subtelomere regions at the end of pseudochromosomes, considering tandem repeat sequences as possible telomere regions based on specific criteria [57]. For instance, tandem repeat sequences located within 20 kb of the end of a contig or pseudochromosome, with a 5–15 bp tandem unit and over 75% identity, were treated as potential telomere regions. For subtelomere regions, we set a minimum length requirement (> 20 kb) for the total tandem repeat sequence. Besides, we filtered tandem repeat sequences to determine potential centromere region based on features such as tandem repeat unit (≥ 100 bp), repeat times (≥ 100), and position on the chromosome according to a previous study focusing on *R. palmatum* [64].

Protein-coding genes in the *R. palmatum* genome were predicted with multiple approaches, including *de novo* gene prediction, homology-based prediction, and RNA-seq annotation. We utilized various programs such as Augustus v3.2.3 (https://github.com/Gaius-Augustus/Augustus), Genescan v1.0 [65], GlimmerHMM v3.04 (https://github.com/mpertea/GlimmerHMM), GeneID v1.4.4 (https://github.com/guigolab/geneid) and SNAP v2013.11.29 (https://github.com/KorfLab/SNAP) for *de novo* gene prediction. For the homology-based prediction, protein-coding genes from related species (i.e., *Fagopyrum tataricum*, *Rumex hastatulus*, *Beta vulgaris*, *Hylocereus undatus* and *Simmondsia chinensis*) were mapped to the *R. palmatum* genome via tBLASTn v2.13.0 [66], and gene structure prediction were performed using GeneWise v2.4.1 (https://www.ebi.ac.uk/seqdb/confluence/display/THD/GeneWise). In the RNA sequencing-assisted prediction, RNA sequencing data from different tissues were mapped to the genome sequence of *R. palmatum* to identify exons and splice positions using Hisat2 v2.2.1 with default parameters [67]. Finally, an integrated non-redundant set of reference genes was constructed via EVidenceModeler v1.1.1 (https://github.com/EVidenceModeler/ EVidenceModeler) based on the prediction results and further updated by PASA v2.4.1 (https://github.com/PASApipeline/PASApipeline). Functional annotation of the final protein-coding genes was performed by searching against public databases (i.e., SwissProt, Nr, Pfam, KEGG, Trembl, and InterPro) with an e-value threshold of 1 × 10^− 5^.

In addition, predictions for the gene structure of tRNA, rRNA, and other non-coding RNAs were also made in the *R. palmatum* genome. The tRNA genes were predicted by tRNAscan-SE v1.3.1 (https://github.com/UCSC-LoweLab/tRNAscan-SE) with default parameters, while rRNAs were identified using RNAmmer v1.2 (https://services.healthtech.dtu.dk/service.php? RNAmmer-1.2). For non-coding genes related to miRNAs and snRNAs, we utilized INFERNAL v1.1 (https://github.com/EddyRivasLab/infernal) to spot potential genes through a search against the Rfam database v1.1 with default parameters.

Comparative genome and phylogenetic analysis.

We determined the recent whole genome duplication (WGD) event in *R. palmatum* and its counterparts (e.g., *F. tataricum*, *Haloxylon ammodendron*, *B. vulgaris*, *Spinacia oleracea*, and *Vitis vinifera*) based on the distribution of synonymous substitutions per site (*K*s) within homologs. The software WGDI v0.6.4 was used to identify synteny blocks among these species [68], and the *K*s values between colinear genes were calculated with the YN00 algorithm. Furthermore, the fourfold degenerate synonymous site (4DTv) of each gene pair within these blocks was estimated using a public script to detect WGD events [69]. We then visualized the *K*s distribution of homologous blocks via WGDI and NGenomeSyn v1.3.8 [70] in dot and block plots, respectively. Additionally, DupGen_finder v20190425 (https://github.com/qiao-xin/DupGen_finder) and MCScanX [71] with default parameters were utilized to identify gene duplication patterns by referencing the *V. vinifera*’s genome [72].

The program OrthoFinder v2.4.0 [73] was utilized to infer the orthologs among the *R. palmatum* genome and other sixteen species (Table S1) with the parameters “-M msa -S blast -A muscle -T fasttree”. Expansion and contraction of gene families were detected using the software CAFÉ v5.0.0 [74], considering significance at 0.05 after excluding families with over 100 members. The function enrichment of these expanded and contracted gene family was explored with the R package *ClusterProfiler* v4.2.1 [75].

A supergene alignment matrix was created by concatenating a total of 141 strictly single-copy (SSC) orthologous genes shared among 17 species, which was aligned using MUSCLE with default parameters. For mostly single-copy gene sets (MSC), we carefully selected 344 orthologous groups, ensuring that each group contained one representative member from every species and was present in at least 14 species (representing over 80% of the total). These groups were then concatenated into a comprehensive supergene matrix. To address potential errors arising from sparse gene sampling, we also retrieved 1,885 and 6,128 orthologous groups with one to three and one to twelve copies per species, respectively, designating them as low-copy 3 (LC3) and low-copy 12 (LC12) gene sets. Subsequently, for SSC ad MSC, we performed phylogenetic analysis via RAxML v8.2.12 [76] using partitioned sequences identified by PartitionFinder v2.1.1 (https://www.robertlanfear.com/partitionfinder/) with 1000 rapid bootstrap replicates, with *Oryza sativa* serving as the outgroup. For LC3 and LC12, we utilized astral-pro v1.15.1.3 [77] to infer the coalescent phylogenetic relationships, leveraging independent OG trees generated by IQtree. The nodes of these trees were collapsed based on different bootstrap value thresholds using newick_utils v1.6 (https://github.com/tjunier/newick_utils). Divergence time was estimated using the MCMCTREE method implemented in PAML v4.9j (https://github.com/abacus-gene/paml). We referenced three calibration points available on TimeTree (http://www.timetree.org/) during divergence time estimation: (i) the emergence time of tricolpate pollen in core eudicots (ca. 125 million years ago, Mya); (ii) the emergence time of seed in crown Caryophyllales species (ca. 83.5 Mya), and (iii) the divergence time between monocots and eudicots (ca. 142.1–163.5 Mya). Finally, the phylogenetic trees were visualized using the R package *ggtree*.

Identification of candidates involved in the biosynthesis pathway of active compounds and its expression pattern in rhubarb.

Genes related to anthraquinone and flavonoids biosynthetic pathways were downloaded from KEGG database (Table S2). A BLASTn analysis was subsequently performed with a stringent e-value ≤ 1 × 10^− 20^. These candidate genes were then compared against all genes of *A. thaliana* and *Glycine max*, filtering out those with less similarity to their respective counterparts.

Given the incomplete understanding of the polyketide pathway in anthraquinone biosynthesis, we focused on identifying potentially relevant active gene families. These include PKS, PKC and PKR involved in octaketide biosynthesis, CYP for oxidation, UGT for glycosylation, and OMT for *O*-methylation. We focused on genes bearing specific domains: PF00067 (heme domain for CYP), PF00201 (UDPGT domain for UGT), PF00891 or PF01596 (OMT2 or OMT3 domain for OMT), and PF00195 and PF02797 (CHS_N and CHS_C domain for PKS), sourcing them from PFAM annotation result with an e-value cutoff of 1 × 10^− 3^. Coding sequences were matched against the final assembled genome using BLASTn to detect any potentially missed genes. In addition, we retrieved perakine reductase in *Rauvolfia serpentina* (AY766462.1) and olivetolic acid cyclase in *Cannabis sativa* (JN679224.1) from GenBank to predict PKR and PKC, respectively [23]. These seed sequences were searched against *R. palmatum* proteins using BLAST, setting an e-value threshold of 1 × 10^− 5^. To predict possible regulation networks among these genes, we used iTAK v1.8 to tally transcript factors in *R. palmatum* genome with default parameters [78]. Genes involved in specific biosynthetic processes often clustered together on chromosomes. Accordingly, we explored the potential biosynthetic gene clusters (BGCs) in *R. palmatum* using plantiSMASH (https://github.com/plantismash/plantismash) with default parameters [79].

In order to construct phylogenetic trees for gene families, we gathered representative genes from the Cytochrome P450 Homepage (https://drnelson.uthsc.edu/) and UGT Nomenclature Committee website (https://labs.wsu.edu/ugt/), two well-studied gene families encompassing multiple clades (Table S3a, b). We also downloaded *OMT* gene family members reported by Uchida et al. [80] from the KEGG database (Table S3c). For the *PKS* gene family, we retrieved *PKS* sequences of species belonging to Caryophyllales and other anthraquinone accumulation species (Table S3d) [81]. These sequences were then merged, aligned with *PKS* members from *R. palmatum* by MUSCLE [82], and trimmed by Gblock v0.91b with default parameters [83]. Phylogenetic tree were constructed from the trimmed sequences using IQ-tree v2.2.0 [84]. Different trees were rooted in the following clade based on previous studies: CYP51 (CYP), UGT85 (UGT), the node of the CCoAOMT and COMT (OMT) branches, and the branch of PKS sequences of mosses and ferns (PKS). The distribution of PKS members of *R. palmatum* on the pseudochromosome was visualized by MapChart v2.3.2 [85].

The clean reads derived from RNA-seq data were mapped to the assembled genome with Hisat2 under its default parameters [86]. From the initial set, we retained 21 samples with high alignment rate > 80%, comprising 5 roots, 6 stems, 6 leaves, and 4 flowers for subsequent analyses. To assemble transcripts and determine gene expression levels, we employed the PrepDE.py3 script (https://github.com/gpertea/stringtie/blob/master/prepDE.py) and StringTie v2.1.7 [87]. We utilized the R package *DESeq2* v1.36.0 [88] to filter out differentially expressed genes (DEGs) based on the following criteria: |Log_2_(fold change)| ≥ 1, “a false discovery rate (FDR) < 0.05, and average read count > 1 for each sample.

## Results

### Genome assembly and annotation

**Fig. 1:**
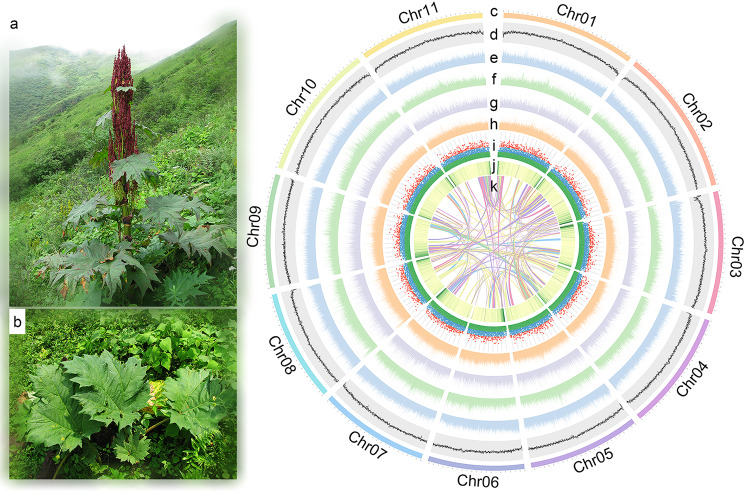
Morphological and genomic characteristics of *Rheum palmatum*. The 200 kb sliding window analyses were carried out to indicate the genome characteristics in (d-h, j) at 50 kb steps. (**a**) morphological characteristics of *R. palmatum* (**b**) leaf morphological characteristics of *R. palmatum* (**c**) eleven pseudochromosomes of *R. palmatum* (**d**) GC% density (**e**) density of all repeat elements; (**f**) density of Ty3/gypsy; (**g**) density of Ty1/copia; (**h**) density of all LTR elements; (**i**) insertion time of LTR elements; (**j**) gene density; (**k**) chromosome synteny

Our genome survey of *R. palmatum* revealed a genome size of approximately 2.8 Gb, with a heterozygosity of 0.32% and a repeat sequence of 82%. By combining HiFi (58 Gb, 21×), ONT (62 Gb, 22×), Illumina (402 Gb, 144×) and Hi-C (347 Gb, 124×) sequencing data (Table S4), we assembled a ca. 2.8 Gb genome with a contig N50 of 9.88 Mb and a scaffold N50 of 259.19 Mb (Fig. 1; Table 1 and S5). Over 99.9% of the sequences were anchored into 11 pseudochromosomes, consistent with our previous study [64] and estimates from *k*-mer frequency method (Figures S1). The assembly’s quality was further confirmed by BUSCO and LAI analyses, which showed high coverage of core genes and assembly accuracy (Table 1). Additionally, the high consensus QV score (46.41, 2.29 × 10^− 5^) also indicated the high assembly accuracy of *R. palmatum* genome.

**Table 1:** Statistics of genome assembly of *R. palmatum*

Item		Result
Estimation of genome	Predict genome size (*k*-mer)	2.8 Gb
	Heterozygosity	0.32%
	Estimate GC%	42.21%
	Repetitive%	82.96%
Genome assembly statistics	Assembly genome size	2.805 Gb
	GC%	41.46%
	Contig number	577
	Contig N50	9.88 Mb
	Scaffold number	18
	Scaffold N50	259.19 Mb
Assemble quality	LTR Assembly Index (LAI)	20.23
	QV score	46.41
	Illumina mapping ratio	99.28%
	Illumina coverage	99.98%
BUSCO	Complete (C)	1531 (94.8%)
	Single-copy BUSCOs (S)	1395 (86.4%)
	Duplicated BUSCOs (D)	136 (8.4%)
	Fragmented BUSCOs (F)	18 (1.1%)
	Missing BUSCOs (M)	65 (4.1%)
	Total BUSCO groups	1614 (100.0%)

**Fig. 2:**
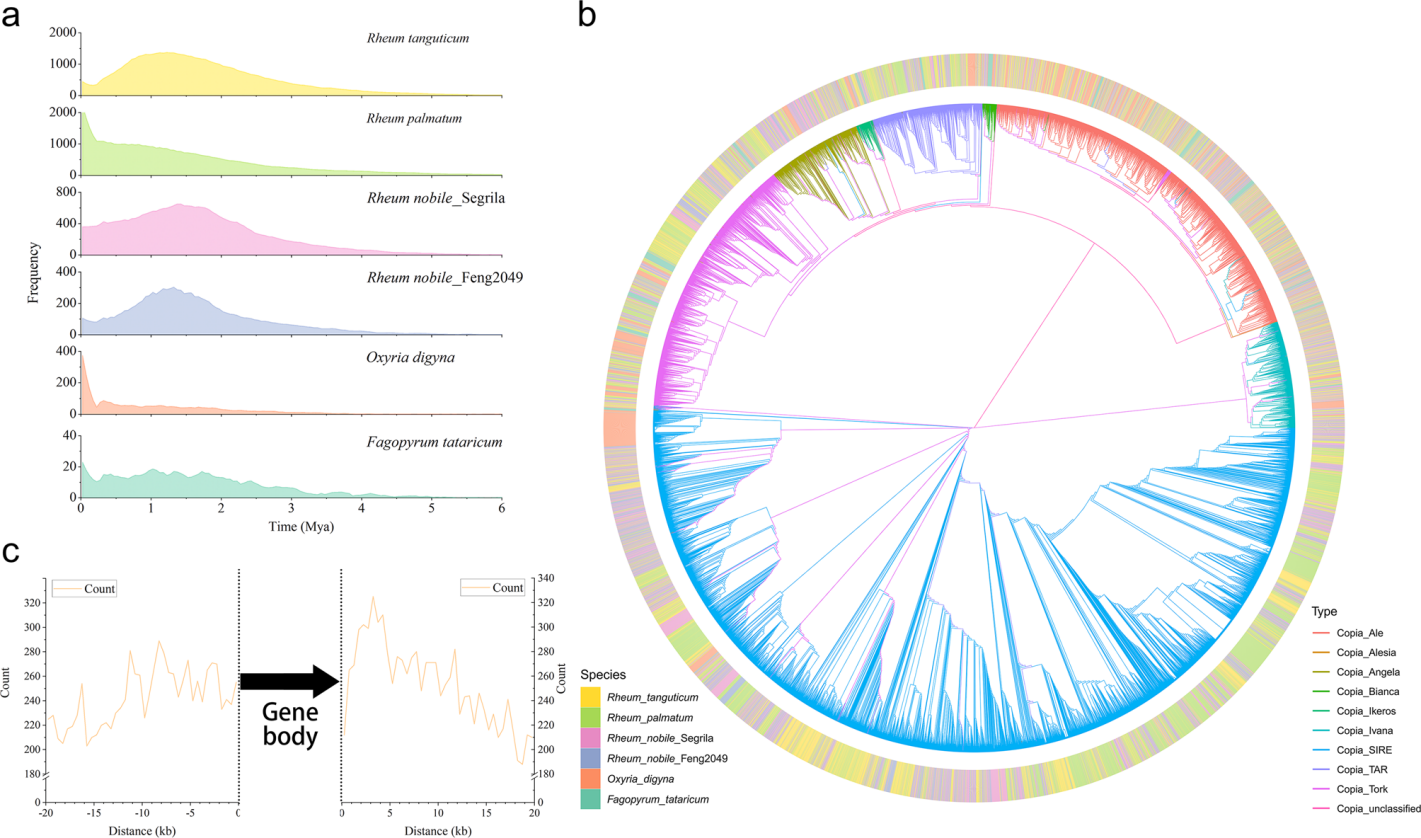
The evolution of LTR elements in the *R. palmaum* genome (**a**) Comparison of LTR insertion time among six Polygonaceae species (**b**) The phylogenesis of subclass Copia members of *Fagopyrum tataricum* (dark green), *Oxyria digyna* (orange), *Rheum nobile* Feng2049 (dark green), *R. nobile* Segrila (dark blue), *Rheum tanguticum* (gold) and *R. palmatum* (light green) (**c**) The distribution of distance between LTR insertion site and protein-coding gene loci

Our integrative approaches identified approximately 2.43 Gb (86.92%) of repeat sequences in *R. palmatum* (Table S3), with Class I and Class II transposon elements accounting for 2.00 Gb (71%) and 0.14 Gb (5%), respectively. LTR element, especially Copia and Gypsy, were the most abundant and contributed significantly to the genome expansion of our focal species. We also observed recent burst events in specific LTR families, such as Gypsy/Tekay, Copia/SIRE and Gypsy/CRM (Tables S7-S8, Fig. 2b and S3), suggesting a possible genus-specific expansion. Additionally, the insertion of LTRs did not show any preferences upstream from the translational start site, while the 3 kb downstream was inserted more frequently than the other sites (Fig. 2c).

A total of 30,480 protein-coding genes were annotated in the *R. palmatum* genome (Table 2 and S9), with an uneven distribution and a majority located near the terminal of pseudochromosomes (Fig. 1j). Most protein-coding genes (94.20%) enabled to get at least one hit against the eight public databases (Table S10), which were mainly associated with carbohydrate metabolism, translation, and biosynthesis of secondary metabolites in KEGG annotation (Figure S4a), while the GO annotation showed an abundance of these genes involved in translation, integral component of membrane, and ATP binding (Figure S4b).

**Table 2:** Statistics of predicted protein-coding genes in *R. palmatum*

Item	Result
Number of coding genes	30,480
Average length of genes	2895.3 bp
Average length of CDSs	1094.6 bp
Median length of CDSs	897 bp
Number of exons per gene	4.99
Average length for single exon	219.4 bp
Average length for single intron	451.5 bp

In addition to the genes encoding proteins, our analysis also anticipated genes that do not encode proteins. In total, we identified 2,765 tRNA, 2,811 rRNA, 83 miRNA, and 1,113 snRNA. Notably, CD-box snRNA and 5 S rRNA were the most abundant types of snRNA and rRNA, respectively (Table S11). Furthermore, within the genome of *R. palmatum*, we also predicted potential regions of telomeres and subtelomeres. Specifically, RpChr06, RpChr08, RpChr09, and RpChr11 displayed four potential telomeres at one end, with lengths ranging from 1.8 kb to 6.5 kb (Figure S5). Interestingly, three of them exhibited a repeat pattern of TTTAGGG, while one displayed a novel repeat pattern (TTTGGGG) (Table S12). In addition, our screening process identified eleven potential subtelomeres and two centromere regions distributed across different pseudochromosomes in *R. palmatum* (Table S12). However, it’s worth noting that no evident telomere or subtelomere regions were detected in other remaining pseudochromosomes.

### Comparative genomic and phylogenetic analysis

We conducted a comparative analysis of the genomes of *R. palmatum* alongside sixteen other representative species, spanning a broad range of plant diversity. Across these genomes, a total of 501,687 genes were clustered into 33,309 gene families, averaging 15.1 genes per family (Table S13a). Notably, 141 strictly single copy families were shared by 17 species, while 324 gene families were uniquely present in *R. palmatum* (Tables S13b and S13c). Function enrichment analysis indicated that genes specific to *R. palmatum* were predominantly associated with processes like rRNA processing, chloroplast fission, tRNA processing, glycolysis/gluconeogenesis, and endocytosis (Figure S6).

**Fig. 3:**
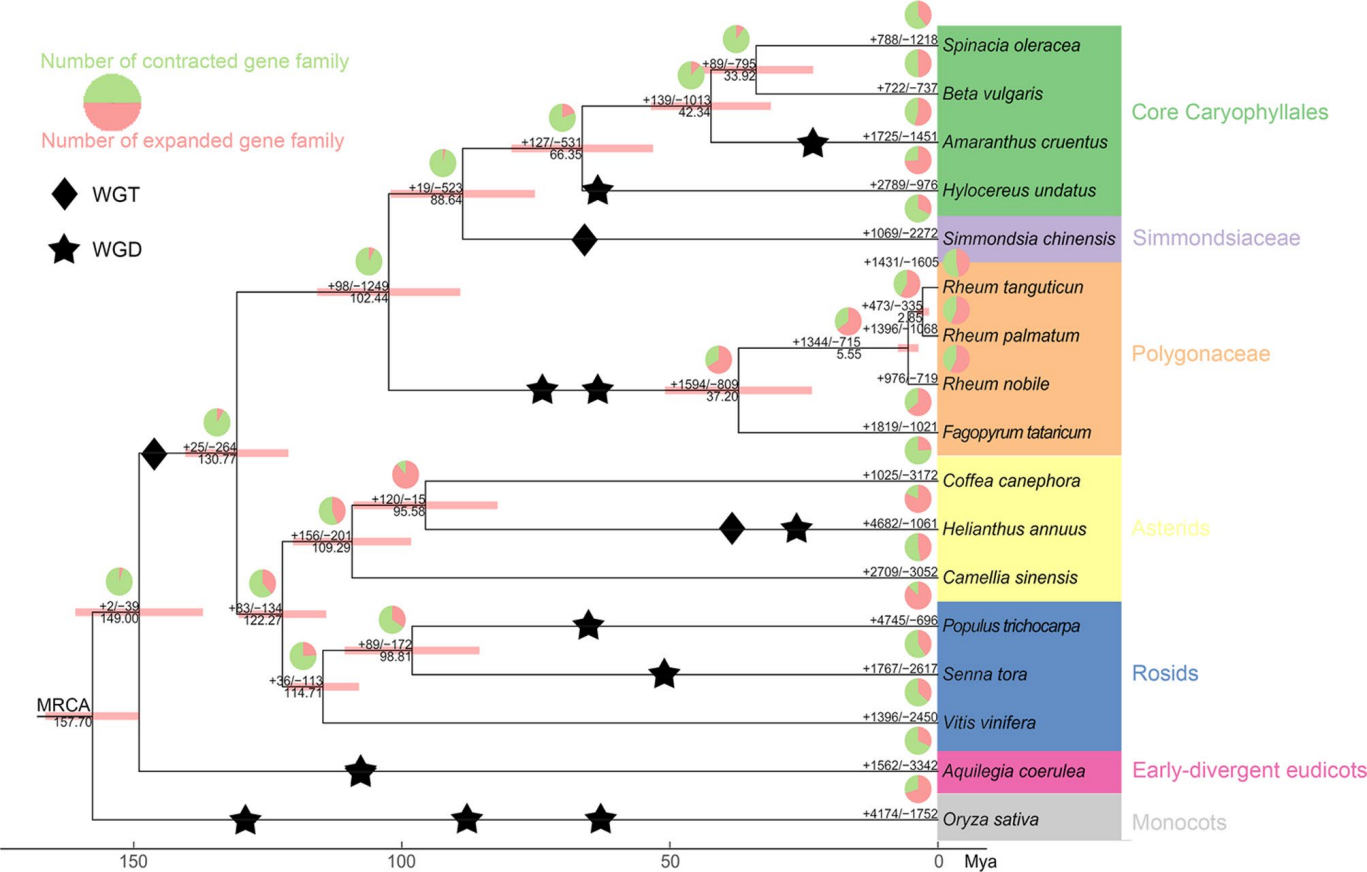
The maximum likelihood-based phylogenetic relationship, the estimate divergent time, the whole genome duplication, and the number of expanded and contracted gene family of sixteen representative species and *R. palmatum*. Pie in red and green meant the ratio of expanded and contracted gene family, a diamond and star indicated a whole genome triplication (WGT) and WGD event, number in brackets under phylogenetic tree branch was the estimated divergent time, and red bands indicated 95% highest posterior density of each divergent event

A phylogenetic tree constructed using the 141 strictly single-copy (SSC) genes revealed a non-canonical relationship (Fig. 3). The rosids and asterids formed a clade with Caryophyllales as their sister group, contradicting the established topology in APG IV [89].To further investigate the phylogeny among rosids, asterids, and Caryophyllales, we incorporated additional gene sets into our analysis. Notably, the topology observed for the mostly single-copy (MSC, comprising 344 orthologous groups) exhibited consistency with that of SSC genes (Figure S7a, S7b). Furthermore, the implementation of a coalescent approach, relying on low-copy gene sets, provided further evidence supporting the position of Caryophyllales as the sister group to rosids and asterids (Figure S7). Estimations of divergence time suggested that *R. palmatum* diverged from *F. tataricum* around 37.2 Mya (95% highest posterior density (HPD): 24.1–51.58 Mya), and the split between Polygonaceae and other Caryophyllales species occurred ca. 102.44 Mya (95%HPD: 89.26–115.97 Mya) (Fig. 3). Accordingly, the corresponding substitution rate was 5.95 × 10^− 9^ substitution/site/year.

Based on this phylogenetic framework, we finally identified 473 expanded and 335 contracted gene families in RPC (Fig. 3). GO enrichment analysis shed light on the biological significance of these changes: expanded gene families in RPC were enriched in functions such as photosynthesis, cell surface receptor signaling pathway, thylakoid, polysaccharide binding, and terpene synthase activity, whereas the contracted genes were associated with the processes like calcium ion binding, apoplast, and lignin catabolic process (Figure S8). KEGG enrichment analysis further highlighted that these expanded gene families were mainly involved in transcription machinery, photosynthesis proteins, and RNA polymerase, while the contracted genes were related to oxidative phosphyrylation and biosynthesis of various plant secondary metabolites (Figure S8). Interestingly, despite a reduction in genes annotated as cytochrome P450, KEGG enrichment suggested an expansion of gene families involved in metabolism of xenobiotics by CYP450 and drug metabolism by CYP450, pointing to specific gene duplications unique to RPC.

### Genome evolution and duplication

**Fig. 4:**
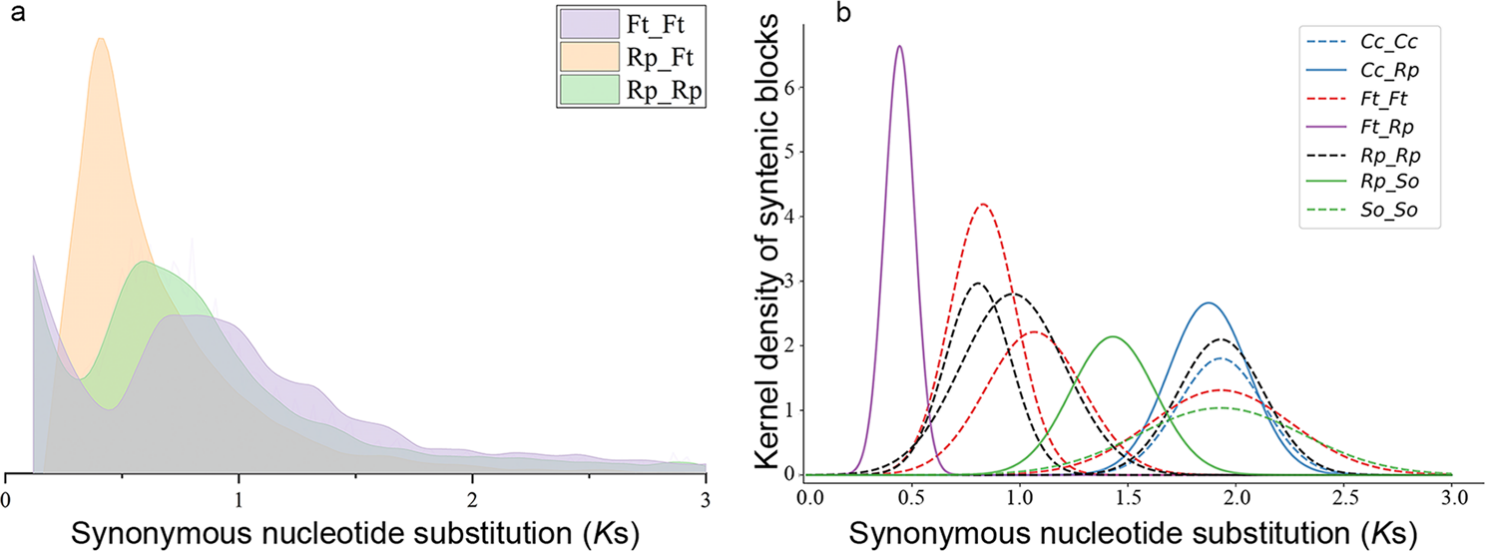
(**a**) *K*s distribution of best hit of gene pairs between *F. tataricum* and *R. palmatum* and within these species. *K*s value less than 0.02 was removed as they corresponded to recent tandem duplication gene pairs (**b**) *K*s distribution of synteny gene pairs between *Coffea canephora* (Cc), *Fagopyrum tataricum* (Ft), *Spinacia oleracea* (So), and *R. palmatum* (Rp) and within these species (dashed line). Different polypoid events were drawn separately

To infer the evolutionary relics from polyploidization in *R. palmatum*, we examined the distribution of synonymous substitution rates per gene (*K*s) and 4DTv between collinear paralogous genes. After analyzing the median *K*s distribution within synteny blocks of the selected genome, we observed a shared *K*s peak at ca. 2.0 among all species, confirming an older γ paleopolyploidy event to core eudicots (Fig. 4 and S9). Additionally, two species-specific *K*s peaks in *R. palmatum* and *F. tataricum* indicated that these species underwent two polyploidization events at ca. 81.2 Mya and ca. 67.7 Mya, respectively (Fig. 3). The 4DTv analysis corroborated these findings, with a shared peak at ca. 0.65 across multiple species corresponding to the γ paleopolyploidy event, and an additional 4DTv peak was detected in *S. chinensis*, confirming one independent recent polyploidization event in this species (Figures S10-S11).

**Fig. 5:**
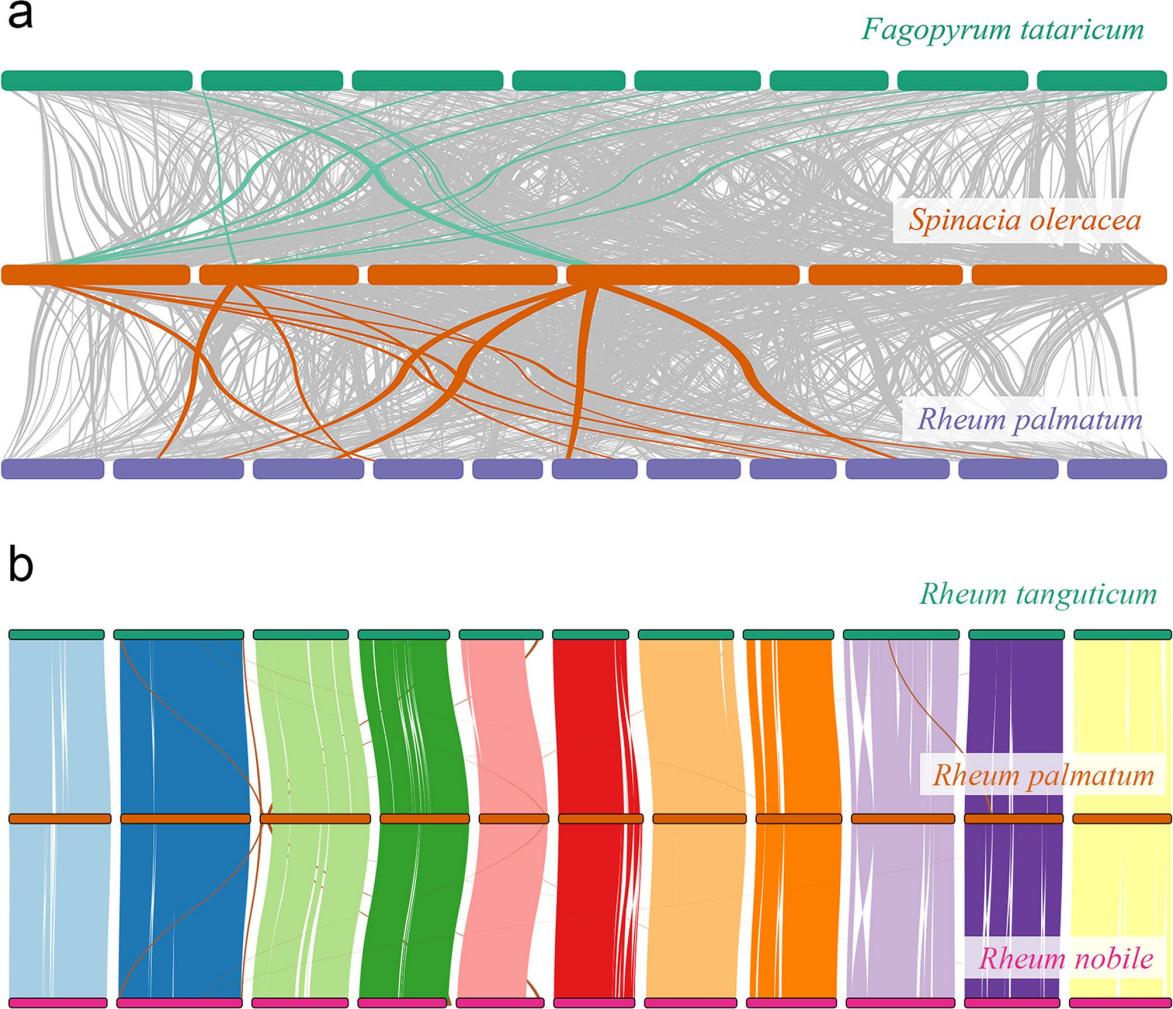
The collinear relationship among different genomes (**a**) *F. tataricum* vs. *S. oleracea* vs. *R. palmatum* (**b**) *R. tanguticum* vs. *R. palmatum* vs. *R. nobile*

Our collinearity analyses involving the genomes of *B. vulgaris*, *H. undatus*, *S. oleracea*, *V. vinifera* and *R. palmatum* provided compelling evidence of polyploidization in *R. palmatum*. We totally identified 823 syntenic blocks encompassing 10,767 genes, accounting for 35.3% of the predicted genes within the *R. palmatum* genome (filtering with *p* < 0.2). Comparisons with other representative genomes revealed 853 to 1151 syntenic blocks, and for each genomic region in *V. vinifera*, *B. vulgaris*, *S. oleracea*, and *Coffea canephora*, we typically detected four matching regions in *R. palmatum* with similar divergence levels and identified 1:4 syntenic depth ratios in all three species pairs (Fig. 5a and S12-15). Furthermore, fragmental polyploidy relic exhibited 2:4 chromosomal relationships in most chromosomes of *H. undatus* and *R. palmatum* genomes (Figure S16). In contrast, the synteny blocks between *F. tataricum*, *R. nobile*, *R. tanguticum*, *Oxyria digyna* and *R. palmatum* displayed a 1:1 ratio (Figures S17-S19), confirming that these sequenced Polygonaceae species shared two consecutive polyploidization events. These lines of evidence strongly suggested that Polygonaceae species underwent two whole genome duplication events.

Chromosome rearrangement is a driving force in genome evolution and often contributes to speciation. In our comparison of the *R. palmatum* genome with *R. tanguticum*, we identified 74 inversions, 15 translocations, and 30 duplicated fragments (Fig. 5b, Table S14a). Genes located within duplicated regions were enriched for functions related to in porphyrin metabolism, ADP binding, kinase inhibitor activity, and acetyltransferase activity, whereas genes suffered rearrangement were implicated in hydrolase activity/hydrolyzing *O*-glycosyl compounds (Table S14b).

We also investigated the origin of novel genes in *R. palmatum* through various duplication modes: disperse duplication (DSD), transposed duplication (TRD), proximal duplication (PD), tandem duplication (TD) and whole genome duplication (WGD). Our analysis using DupGen_finder and MCScanX revealed that TRD (9,306, 30.5%) was the most prevalent duplicated mode in *R. palmatum*, followed by WGD (7,995, 26.2%) and DSD (7,122, 23.4%). The contribution of PD (1,377, 4.5%) and TD (1,760, 5.8%) to novel genes was limited. Notably, the proportion of genes originating from WGD was higher in the *Rheum* (e.g. 26.2% in *R. palmatum*, 46.8% in *R. nobile* Segrila, and 55.1% in *R. tanguticum*), suggesting that the two rounds of paleopolyploidy significantly influenced the genome evolution in *R. palmatum* and its relatives (Table S15).

### Biosynthesis of bioactive ingredients and expression patterns in rhubarb

Anthraquinones, flavonoids and tannins are the primary bioactive compounds found in rhubarb. Through homology searching, we identified genes related to the biosynthesis of these compounds (Figures S20-S21). Our findings indicated that 97 genes participated in the biosynthesis of catechin, gallic acid, and other flavonoids, while 54 genes were homologs to anthraquinone biosynthesis genes (Table S16). Specifically, the biosynthesis of stilbene, isoflavonoids, and catechin/anthocyanin utilized a minimum of five, twenty-six, and thirty genes, respectively. Notably, Four CHS genes and four CHS-like genes were annotated as the key enzyme for flavonoid biosynthesis, and one stilbene synthase (STS, Rh_pal_11G013580.1) was screened for the biosynthesis of stilbenes in rhubarb. In the biosynthesis of isoflavonoids, CYP93C and CYP71D9 had no homologs in the genome of *R. palmatum*, and only 14 genes showed similarity to GmCYP81E, suggesting that different genes could catalyze related reactions.

Anthraquinone is a marker metabolite in rhubarb, and its biosynthesis can be divided into four pathways: shikimate, MEP, MVA and polyketide. We identified a total of 18, 13 and 17 enzyme genes for the first three pathways, respectively. Notably, in the shikimate pathway, the rate-limiting enzyme 3-dehydroquinate synthase (DHQS) coding gene had only one gene copy in *R. palmatum*, while other genes, such as 3-deoxy-7-phosphoheptulonate synthase (*DAHPS*, 3 copies), naphthoate synthase (*menB*, 3 copies), and 1,4-dihydroxy-2-naphthoyl-CoA hydrolase (*menI*, 4 copies) had multiple copies. In the MEP pathway, more than two copies were identified for rate-limiting enzymes, i.e. 1-deoxy-*D*-xylulose-5-phosphate synthase (DXS), 1-deoxy-*D*-xylulose-5-phosphate reductoisomerase (DXR), and 4-hydroxy-3-methylbut-2-enyl diphosphate reductase (HMB-PPR). However, we did not observe the presence of 2-C-methyl-*D*-erythritol 2,4-cyclodiphosphate synthase (*ME-cPPs*) in *R. palmatum*. As for MVA pathway, six 3-hydroxy-3-methylglutaryl-CoA reductases (*HMGRs*) were screened and differentially expressed among organs.

**Fig. 6:**
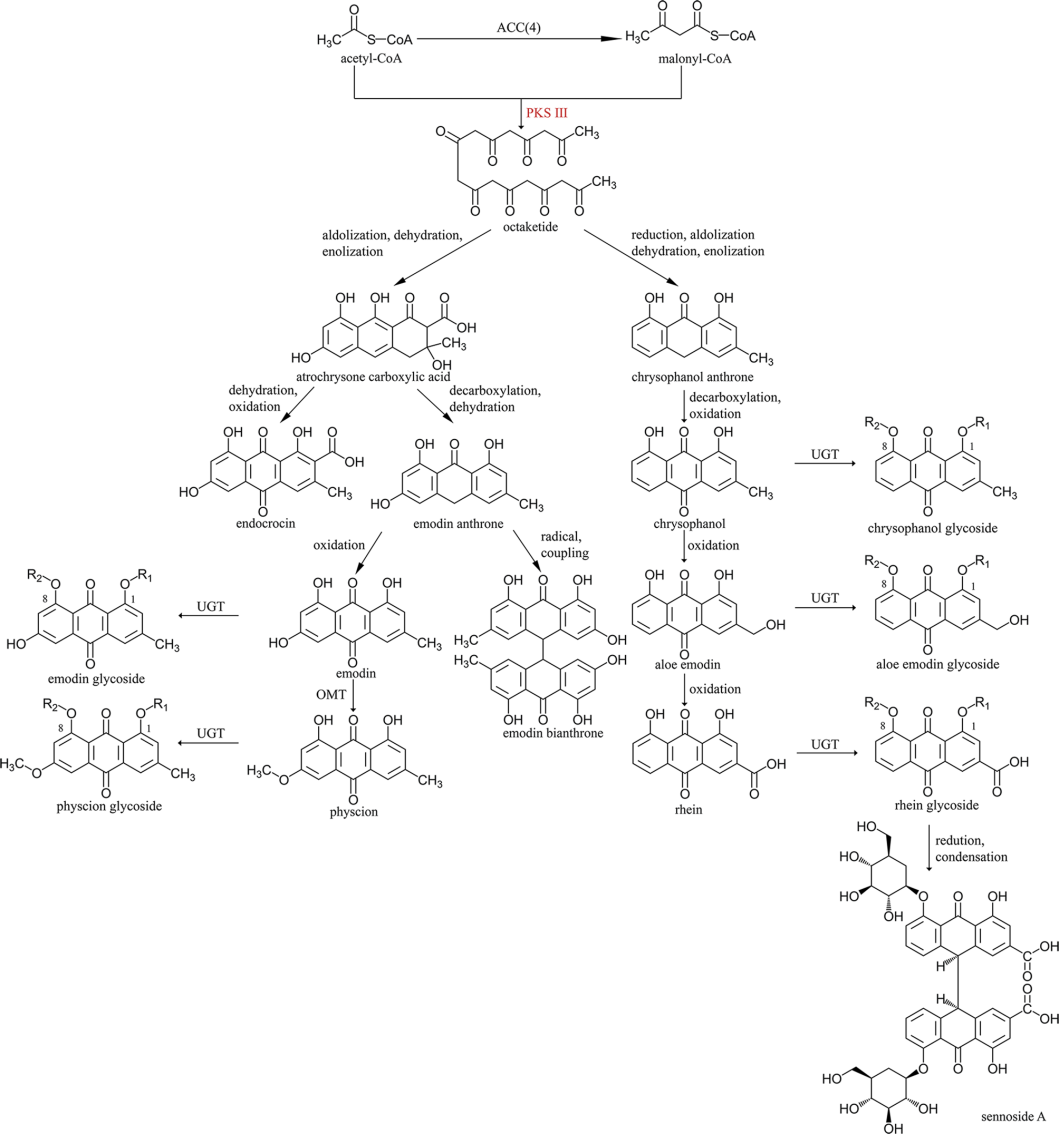
Predicted polyketide pathway of anthraquinone biosynthesis in rhubarb

Given the limited understanding of the polyketide pathway, especially the PKS enzyme (Fig. 6), we explored potential genes involved in bioactive ingredient biosynthesis based on conserved domains. Several gene families were screened, including PKS for octaketide formation, CYP for oxidation, UGT for glycosyl transfer, and OMT for methyl transfer to hydroxyl group. We detected 16, 227, 116, and 31 members for the aforementioned families, respectively (Tables S17-S19, Figures S22-S24). Interesting, some clades, such as CYP76, UGT81, and UGT71, had more members compared to the related species. Nine CCoOMTs and twenty-two COMTs were found and clustered with different representative OMTs, respectively.

**Fig. 7:**
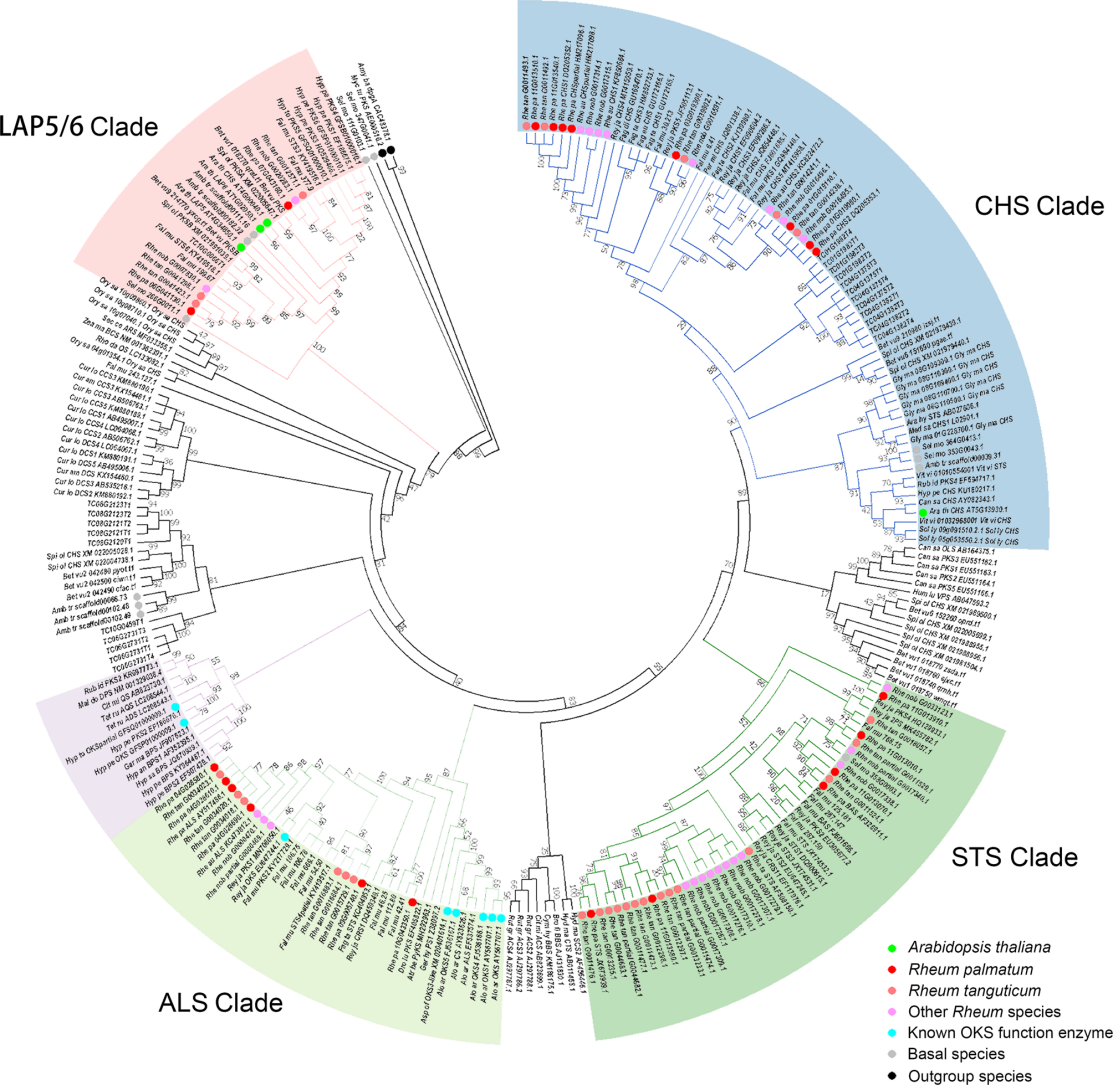
The phylogeny of representative PKS III family members. Dots in different colors represent the source species of PKSs. LAP5/6, CHS, STS, and ALS clade was colored in pink, light blue, dark green and light green background. Dots in different colors represent the source species of PKSs. LAP5/6, CHS, STS, and ALS clade was colored in pink, light blue, dark green and light green background

The PKS gene family members are believed to be pivotal enzymes for plant landing, catalyzing numerous important reactions [90]. In rhubarb, our investigation uncovered 16 PKSs distributed across eight pseudochromosomes (Table S20, Figure S25a). These include two benzalacetone synthase (BASs), three aloesone synthases (ALSs), one LESS ADHESIVE POLLEN 5 (LAP5), and one LAP6 classified based on phylogenetic relations (Fig. 7). The function of OKS enzyme was recently verified in *Re. japonica* [18]. Our analysis focused on screening potential octapeptide synthase (OKS) in *R. palmatum*, with ALSs possibly emerging as the prime candidates. Notably, the conserved sites, such as sites related with catalytic triad (164C-303H-336N), gatekeeper (215F and 265F), product length control (197A), and inner (256L and 338T) between RjOKS and RpALSs were identified, indicating that RpALSs might be responsible for octaketide formation in rhubarb (Figure S26) [90–92]. In addition, we identified seven PKR and six PKC candidates in *R. palmatum* by homologous searching (Table S18). However, the sequence identity for PKC was approximately 28% to the known PKC, specifically olivetolic acid cyclase in *C. sativa* (JN679224). To further explore potential regulators, a whole genome search for transcript factor families was conducted in *R. palmatum*, uncovering 2,379 genes with at least one transcript factor domain (Table S22). The top five families identified were R2R3-MYB (151), AP2/ERF (141), bHLH (140), C2H2 (118), and bZIP (97).

Genes involving in specialized metabolism often cluster together and co-regulated in neighboring chromosomal regions, enhancing their responses to biotic or abiotic stress in evolution. Our predictions in *R. palmatum* revealed 44 potential clusters (Table S23a). Notably, six clusters were associated with terpene biosynthesis, three were correlated with saccharide-polyketide biosynthesis, and four were linked to saccharide biosynthesis. Additionally, clusters linked to polyketide, saccharide-alkaloid, and saccharide-terpene were identified. Of particular interest, the three RpALSs were found adjacent to eleven other genes, including a ligase, a UGT, a kinase, and an IQD transcription factor in cluster 15 (Table S23b). Moreover, on RpChr11, RpBASs (*Rh_pal_11G013010.1* and *Rh_pal_11G013070.1*) were observed to be adjacent to one CYP and one amino oxidase in cluster 39. These genes exhibited high expression levels in leaves (Table S10, Table S23b), suggesting potential co-regulation in benzalacetone biosynthesis within *R. palmatum*.

## Discussion

We assembled a high-quality genome for *R. palmatum*, a source plant for rhubarb, with anchored sequences comprising 99.96% of all scaffolds, leading to one of the best-anchored medicinal plant genomes. A total of 30,480 coding genes and 6,772 non-coding genes were annotated. Our results indicated that two WGD events had profoundly impacted the evolution of Polygonaceae, while transposons contributed to the highly repetitive and complex genomes for *Rheum* species. Although some steps remain unclear, our whole genome screening has shed light on the biosynthesis of bioactive compounds and identified candidates involved in the modification of anthraquinones and flavonoids.

### High repetitive content was common in genus *Rheum*

For angiosperm species, the proportion of repeat regions varies widely, ranging from over 9% in *U. gibba* [93] to approximately 90% in *Zea mays*, *Saccharum spontaneum* and *Paeonia ludlowii* [94–97]. Transposable elements (TEs), which have the ability to move throughout the host genome, constitute one of the primary components of these repetitive sequences. TEs can be categorized into RNA-mediated retrotransposons (class I) and DNA-mediated DNA transposons (class II), depending on their transposition intermediate. Furthermore, the structure of each element provides a basis for their classification into order, superfamily and family. Notably, LTR represents the most prevalent order of TEs in plant genomes. Different LTR families had different activities in different species. In this study, we utilized TEsorter to classify full-length LTRs into families and observed that Gypsy/Tekay, Gypsy/Retand, Gypsy/CRM and Copia/SIRE had higher numbers compared to *R. palmatum* relatives. In addition, we also detected expansions of Copia/Ale, Gypsy/CRM, and Copia/SIRE in *O. digyna* and *R. nobile* Feng2049 (Table S8), respectively, which aligned with the unexpected expansion of Gypsy/Tekay, Gypsy/CRM, and Copia/SIRE in *R. officinale*. The high repeat content is consistently observed in other *Rheum* species, with percentages ranging from 77 to 87% [32, 33, 35, 36]. However, in other closely related species, such as *F. tataricum* (249.3 Mb, 50.96%) [98], *Fagopyrum dibotrys* (0.74 Gb, 68.21%) [99], and *O. digyna* (219.8 Mb, 39.17%), TE proportions are significantly lower than our focal species. Additionally, a recent study has indicated that the high repeat content might be common within the *Rheum* [100].

TEs play multiple roles in genomes, influencing genome size changes and consequently leading to genome evolution and diversity in plants [101, 102]. They are regulated by mechanisms like DNA methylation and chromatin remodeling, ensuing genome stability under normal condition while allowing stress-induced activations [103–105]. Furthermore, TE can indirectly modulate gene expression via non-coding RNA or directly by inserting into gene structure elements [106, 107], potentially rearranging genetic regulatory networks and aiding in environmental adaptation. Given that *Rheum* species usually face abiotic stresses like extreme temperature and high UV radiation, the high TE content was suggested to enhance their tolerance to adverse environments [108]. This, in turn, might facilitate the reconfiguration of their genetic expression network, thereby promoting species diversity and broadening the distribution range of *Rheum* in the Qinghai-Tibet plateau and adjacent areas.

### Transposon burst and WGD in the *R. palmatum* genome

In *R. palmatum*, the distribution of genes in the near-end aligns with that of most plant genomes, but there was no significant variation in GC% along the pseudochromosomes (Fig. 1c), which is in contrast to the observation made in *F. tataricum* genome. Notably, its genomic GC% up to 41.46%, surpassing most sequenced eudicots. We analyzing 1,521 genomes representing 1,105 species from the NCBI genome database, focusing on those with multiple sequencing records. Only thirteen eudicot species exhibited a higher GC proportion than *R. palmatum* (Figure S27, Table S24). Hypotheses explaining GC content variations fall into three categories: selection, mutational biases and GC-biased gene conversion [109]. Higher GC content, like in *Dorcoceras hygrometricum* (42.30%), is speculated to aid environmental adaptation by bolstering genome stability [110]. Yet, in Poaceae genomes where GC content ranged from 38.9 to 49.2% (Table S24), evidence suggests that GC-biased selection is primarily driven by the maladaptive mechanism of biased gene conversion [109]. However, since *Rheum* species are typically self-compatible, as observed in our surveys and reported by Li et al. [34, 35], recombination might not be the primary cause of their higher GC ratio. In addition, GC-biased gene conversion or mutational biases can be discounted due to codon usage bias (Table S25). The results of GC contents in different elements indicated that transposons, especially LTR elements, played a vital role in elevating GC% (Table S26). Furthermore, it was evident that LTR evenly distributed throughout the pseudochromosomes of *R. palmatum*, indicating the LTR insertions might promote the genome stability in our focal species.

Unlike other species, our study indicated that *R. palmatum* maintained a high number of intact LTR elements. TE cleavage left 36% of genome annotated as incomplete Gypsy and 12% as incomplete Copia, remnants of TE bursts in the past million years. It is noteworthy that, despite the consistent expansion of LTR retrotransposons in Polygonaceae species dating back to around ca. 4 Mya, the proliferations of these retrotransposons in *R. palmatum* and *O. digyna* surged to a climax in recent years. When examining its relatives, there has been a preponderance of LTR insertions, especially during the past 1–2 Mya (Fig. 2a). Therefore, the history of LTR insertions elucidated distinct evolutionary trajectories between the *R. palmatum* and its counterparts. Additionally, it was observed that LTR removal was less efficient in *R. tanguticum* compared to *F. tataricum* due to unequal recombination, resulting in LTR accumulation in the *R. tanguticum* genome [35]. The balance between genome stability and LTR accumulation, potentially shaped by LTRs, remains an intriguing unknown mechanism in the *Rheum* and necessitates further study.

We conducted collinearity analyses between *R. palmatum* and *F. tataricum*, *B. vulgaris*, *S. oleracea*, as well as *V. vinifera*, confirming two rounds of WGD in Polygonaceae that previous studies have determined [34, 111, 112]. Estimates of duplication time suggested that the first round of WGD took place ca. 81 Mya (Figs. 3 and 5b), which was after the split between Polygonaceae and its relatives (91–111 Mya) but prior to the dispersal of the crown clade (approximately 75 Mya) [113–115]. Consequently, the first round of WGD was inferred to be shared by all extant Polygonaceae species [116]. In contrast, the second round of WGD was estimated to occur ca. 67 Mya, aligning with the divergence between Eriogonoideae + Polygonoideae and tropically distributed basal taxa, i.e. *Ruprechtia*, *Symmeria*, and *Afrobrunnichia* (67–69 Mya) [100, 117, 118]. Notably, WGD is believed to have played a pivotal role in enabling Polygonaceae species to acquire cold adaptation abilities [119], suggesting that this second round of WGD was instrumental for Polygonaceae to expand its distribution range eastward and northward. However, whether Eriogonoideae species also underwent the second round of WGD remains an open question warranting further investigation [113, 115].

### Potential telomere, subtelomere, and centromere regions in *R. palmatum*

Telomere cap the ends of linear eukaryotic chromosomes, maintaining their long-term stability and proliferation [120]. In plants, the telomere DNA typically presents a classical 7 bp repeat type, “TTTAGGG”. However, empirical studies have observed six to twelve bp repetitive patterns in the species of Iridaceae (TTAGGG) and *Allium* (CTCGGTTATGGG) [121], as well as in *Fagopyrum esculentum* and *F. tataricum*, where the patterns of T(2–4)CGGG, T(2–3)CAGG and T(2–3)CGG were also observed [122]. Our analysis identified four potential telomeres at the ends of RpChr06, RpChr08, RpChr09, and RpChr11, but we failed to discern more features on the remain pseudochromosomes. While one possible reason could be the variable repetitive pattern [122], it underscores the need for telomere-to-telomere (T2T) genome sequencing to obtain a more complete rhubarb genome in future.

Subtelomeres, adjacent to telomeres as satellite-DNA, play critical roles in facilitating meiotic pairing, protecting terminal genes from loss and gain processes, and even regulating gene expression [123]. Our screening detected eleven potential subtelomeres in *R. palmatum*, though no clear signals were evident in the ends of other pseudochromosomes. Interestingly, six of these had a 118–120 base repeat unit, shorter than the telomere-associated satellite DNA found in *Rumex induratus* and *Rumex scutatus* (170 bp), possibly reflecting different evolutionary trajectories. Divergent occurred ca. 23 Mya [124], well before the formation of repeat patterns in *Rumex*.

Identification centromere was more challenging than telomere regions due to their inherent variability. In *R. palmatum*, we determined two potential centromere regions in RpChr10 and RpChr11, respectively. The length of a single repeat unit was as long as 156 bp (Table S12), comparable to centromeres in other plants like *Z. mays* (156 bp) and *Oropetium thomaeum* (155 bp) but shorter than *A. thaliana* (180 bp) [125]. The total repetitive regions spanned 26 kb for each centromere. We failed to detect more potential centromeres in other pseudochromosomes due to gaps in assembly, longer repeat unit, or centromere variation [126–128].

### The biosynthesis of active ingredients in rhubarb

Rhubarb has been utilized for over 1,800 years to alleviate constipation, respiratory distress syndrome, bacterial infection and severe acute pancreatitis, sepsis, and chronic renal failure [14]. Its main active compounds include anthraquinone, flavonoids, and tannins. In this study, we identified 97 and 54 genes responsible for biosynthesizing these compounds or their monomers. Flavonoids can be divided into several groups based on chemical structures. Notably, isoflavonoids, proanthocyanidins, and stilbenes, often overlooked have been proven effective in enhancing human health [129–131]. In the present study, we identified eleven phenylalanine ammonia-lyases (PALs), four cinnamate 4-hydroxylases (4CHs) and ten 4-coumarate-CoA ligases (4CLs) involving in producing common precursors cinnamoyl CoA and p-coumaroyl CoA. CHS-like sequences had fourteen copies, though phylogenetic analysis suggested that five copies were more likely to function as CHS (Fig. 7). In subsequent steps leading to catechin, enzymes had at least three copies, except for anthocyanidin synthase (ANS) and flavonoid 3′-hydroxylase (F3’H), which had single copies *Rh_pal_07G033840* and *Rh_pal_02G057630*, respectively. Interestingly, several enzymes in isoflavonoids biosynthesis had no homologs, such as CYP93C, CYP71D, and HI4OMT. Though the biosynthesis of isoflavonoids is well-documented in Fabaceae [132], we speculate that the recruited enzymes in Polygonaceae may differ due to convergent evolution.

Intriguingly, rate-limited enzymes had multiple copies in all anthraquinone biosynthesis pathways except the shikimate pathway (Figure S20). In this pathway, seven enzymes catalyzing the biosynthesis from 3-deoxy-*D*-arabino-heptosonate 7-phosphate (DAHP) to *o*-succinyl CoA had only one copy. Our analysis uncovered that these enzymes were primarily classified as singletons by DupGen_finder and MCScanX and are thus essential for plant function [133]. Specific enzymes like 3-dehydroquinate synthase (*DHQS*), 3-phosphoshikimate 1-carboxyvinyltransferase (*EPSPS*), and chorismate synthase (*CS*), along with two copies of *DAHPS*, had high expression in stems. In contrast, *o*-succinylbenzoic acid—CoA ligase (*AAE14*) and one copy of *menB* had the highest expression in flowers. In MEP and MVA pathways, several genes exhibited high expression levels in flowers. However, the correlation between expression patterns and anthraquinone accumulation in *R. palmatum* was inconsistent, possibly due to shared biosynthetic steps between anthraquinone and terpenoid in MVA and MEP pathways. Additionally, the first three steps of the shikimate pathway were involved in the biosynthesis of both anthraquinone and tannins’ monomer, gallic acid. No homology of *ME-cPPs* was found, which might be due to assembly limitations in obtain these genes as identified in transcriptome studies of *R. palmatum* [134].

Some steps of anthraquinone biosynthesis in rhubarb remain enigmatic, specifically alizarin biosynthesis and most reactions in the polyketide pathway. It is speculated that one PKS gene family member catalyzes the formation of octaketide. However, RpALS, RpBAS, RpSTS, RpCHS1, and RpCHS2 could not produce corresponding compounds in previous studies [19, 20, 135]. Our whole-genome identification of the PKS gene family in *R. palmatum* yielded sixteen members distributed across eight pseudochromosomes. Surprisingly, despite significant anthraquinone compounds accumulation in rhubarb, PKSs did not experience notable expansion in *R. palmatum* compared to other similar genes in medicinal plants, i.e. CYP725 in *Taxus* [27, 28]. Typically, 10 to 16 PKSs were identified in other representative Caryophyllales species (Figure S25d), except for *Amaranthus cruentus* (4 PKSs) and *Chenopodium quinoa* (34 PKSs). The exon number of PKS genes ranged from two to four, with *Rh_pal_11G0139130.1* as an exception, indicating minimal gene structural variations (Figure S25c).

Previous studies had indicated that mutations at specific sites could alter the substrate preference of members in the PKS gene family. For instance, one amino acid mutation from histidine to glutamine resulted in varied enzyme actions of STS in *Arachis hypogaea* [136]. Moreover, a subtle change in amino acid composition abolished acridone synthase (ACS) activity in *Ruta graveolens* [137], highlighting the crucial role of key amino acid in determining the substrate, products and enzyme activity of PKSs. Recently, Guo et al. confirmed that RjOKS was responsible for octaketide biosynthesis in *Re. japonica* [18]. By compared the sequences of PKS members in *R. palmatum* with RjOKS, we identified that ALS shared identical key amino acids, suggesting a potential similar function. An earlier study postulated that RpALS produced aloesone, a heptaketide, through the conversion of acetyl-CoA and malonyl-CoA [138]. However, the experiment was conducted in prokaryotic cells, which may not fully recapitulate the conditions in eukaryotes. Additionally, recent findings have implicated PKR in naphthoquinone biosynthesis in *Plumbago zeylanica*, hinting at the involvement of multiple coenzymes in anthraquinone biosynthesis [23]. This could explain why the in vivo function of ALS yet to be confirmed and underscores the need for further investigation in eukaryotic expression system, especially in *R. palmatum*, to elucidate the roles of RpALS and other coenzymes.

## Conclusion

Our study had successfully assembled a high-quality genome of *R. palmatum*, shedding light on the evolutionary history of Polygonaceae and the biosynthesis of bioactive ingredients in rhubarb. The two rounds of WGD had profoundly influenced the evolution of *R. palmatum*. RpALSs emerge as promising candidates for anthraquinone biosynthesis, aided by PKR and PKC and downstream modification enzymes like CYP, UGT, and OMT. Furthermore, the genomes of *Rheum* species contain exceptionally high levels of TEs, opening avenues for exploring the regulatory mechanisms of LTR accumulation and their adaptative functions. Our findings establish a foundation for future molecular research on RPC, including high-altitude adaptation, diversification, and gene function studies.

### Electronic supplementary material

Below is the link to the electronic supplementary material.

Supplementary Material 1

Supplementary Material 2

Supplementary Material 3

Supplementary Material 4

Supplementary Material 5

## Data Availability

The datasets supporting the conclusions of this article are included within the article and its additional files. The raw genome and transcriptome sequencing data reported in the present study have been deposited in the National Center for Biotechnology Information (NCBI) database under project number PRJNA719574, PRJNA735904, PRJNA827652, and PRJNA1049137. The assembled genome and the genome annotation are available at figshare database (10.6084/m9.figshare.25495309). And the structure of protein-coding gene can also be retrieved from Additional File 5 (Data S1).

## Abbreviations

RPC: the *Rheum palmatum* complex
TE: Transposable elements
LTR: long terminal repeat retrotransposon
WGD: whole genome duplication event
PKS: polyketide synthase
