# Supplementary material for: A chromosome-level genome reveals genome evolution and molecular basis of anthraquinone biosynthesis in Rheum palmatum
Source: BMC Plant Biol. 2024 Apr 10;24:261. doi: 10.1186/s12870-024-04972-2 (PMC11005207; doi:10.1186/s12870-024-04972-2)
Supplement: Supplementary file 4 — Supplementary Material 4 [file 12870_2024_4972_MOESM4_ESM.docx]

**Title**

A chromosome-level genome reveals genome evolution and molecular basis of anthraquinone biosynthesis in *Rheum palmatum*

**Author information**

Tianyi Zhang^1^, Lipan Zhou^1^, Yang Pu^1^, Yadi Tang^1^, Jie Liu^1^, Li Yang^1^, Tao Zhou^1^, Li Feng^1,^*, Xumei Wang^1,^*

**Corresponding author**

Xumei Wang*

[wangxumei@mail.xjtu.edu.cn](mailto:wangxumei@mail.xjtu.edu.cn)

Li Feng*

[Lifeng007@xjtu.edu.cn](mailto:Lifeng007@xjtu.edu.cn)

**Address**

^1^ School of Pharmacy, Xi'an Jiaotong University, Xi'an 710061, China

**Supplementary Figures**

**Figure S1** Genome size estimation of *Rheum palmatum* by *k*-mer method


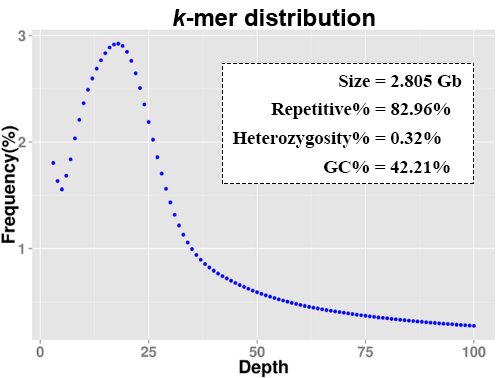


**Figure S2** Signal intensity heat map of the Hi-C chromosomal interaction in *R. palmatum*

**Figure S3** Phylogenesis of subclass Gypsy members of *Fagopyrum tataricum* (dark green), *Oxyria digyna* (orange), *Rheum nobile* Feng2049 (darkgreen), *R. nobile* Segrila (dark blue), *Rheum tanguticum* (gold) and *R. palmatum* (light green)


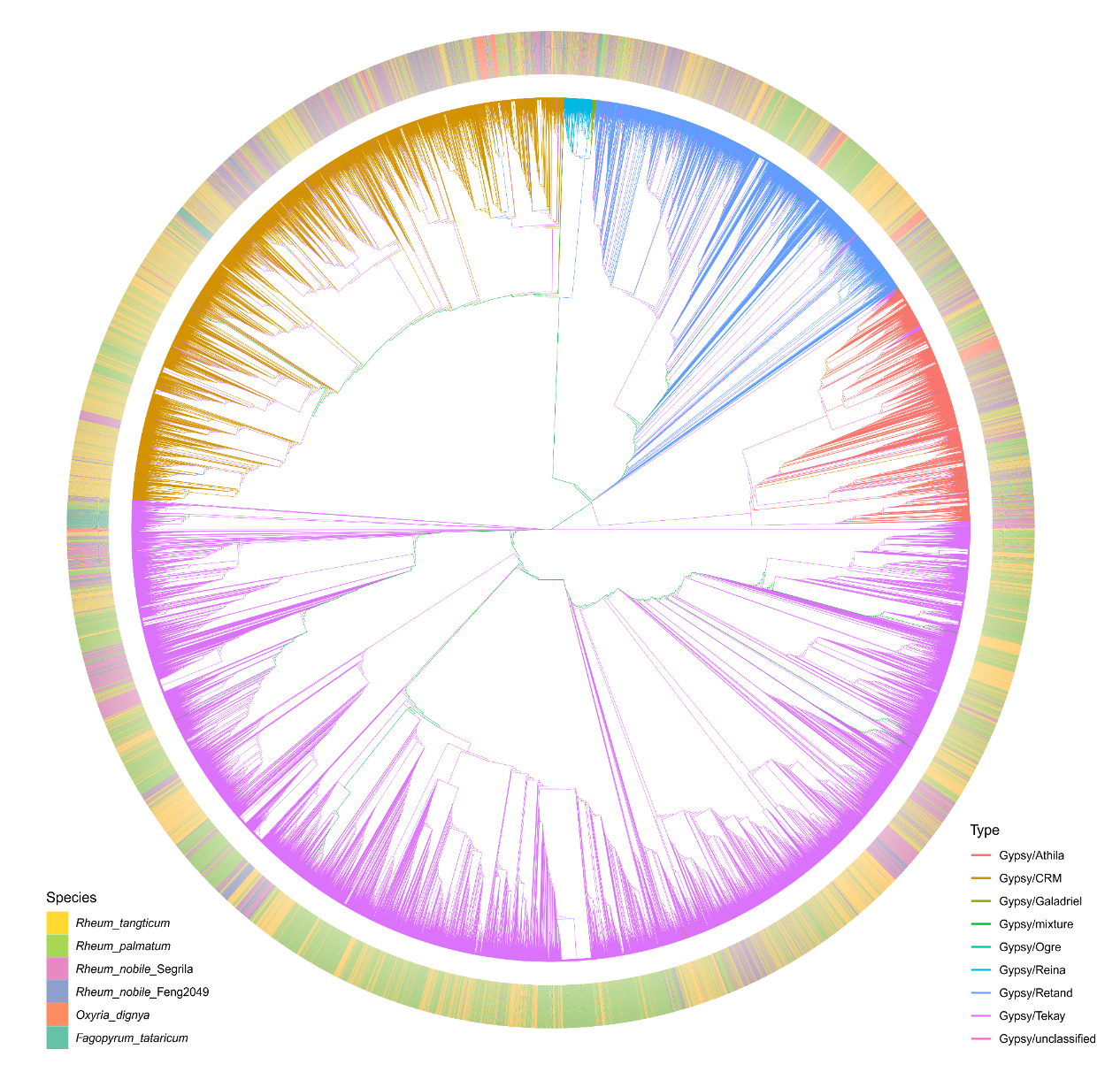


**Figure S4** KEGG (a) and GO (b) annotation of all protein-coding genes in *R. palmatum*


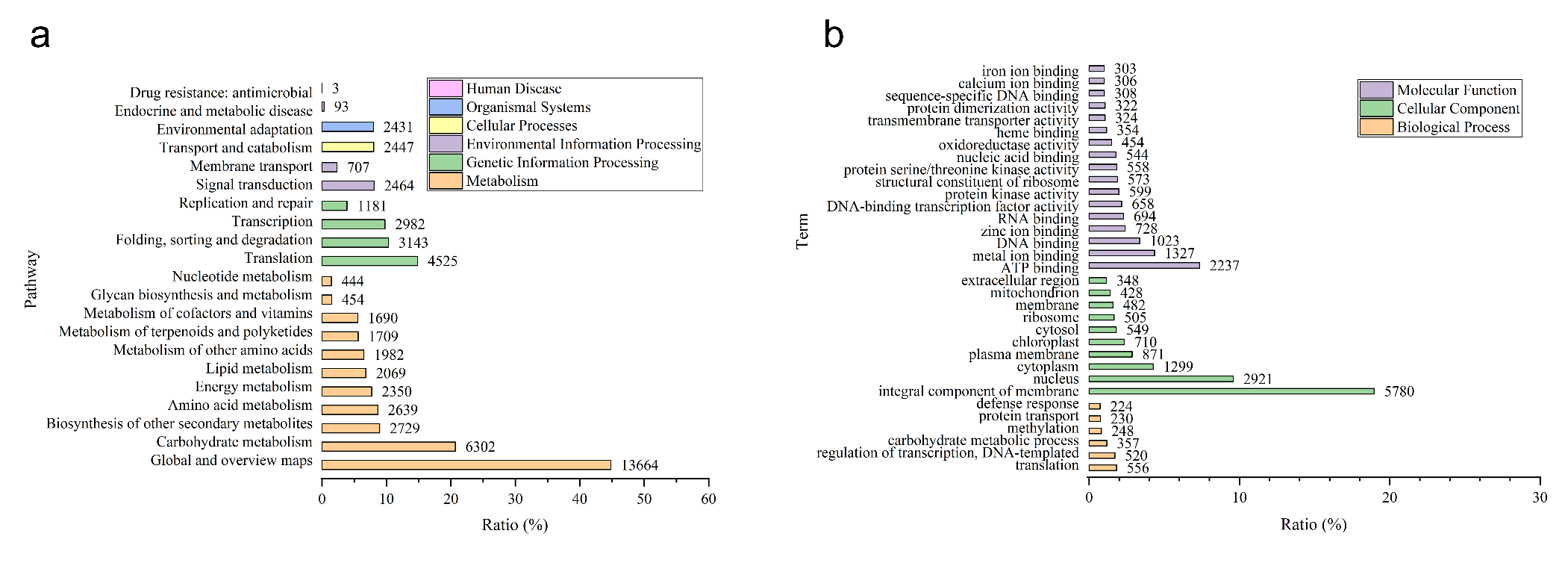


**Figure S5** Prediction of telomere and centromere regions of pseudochromosome of *R. palmatum*


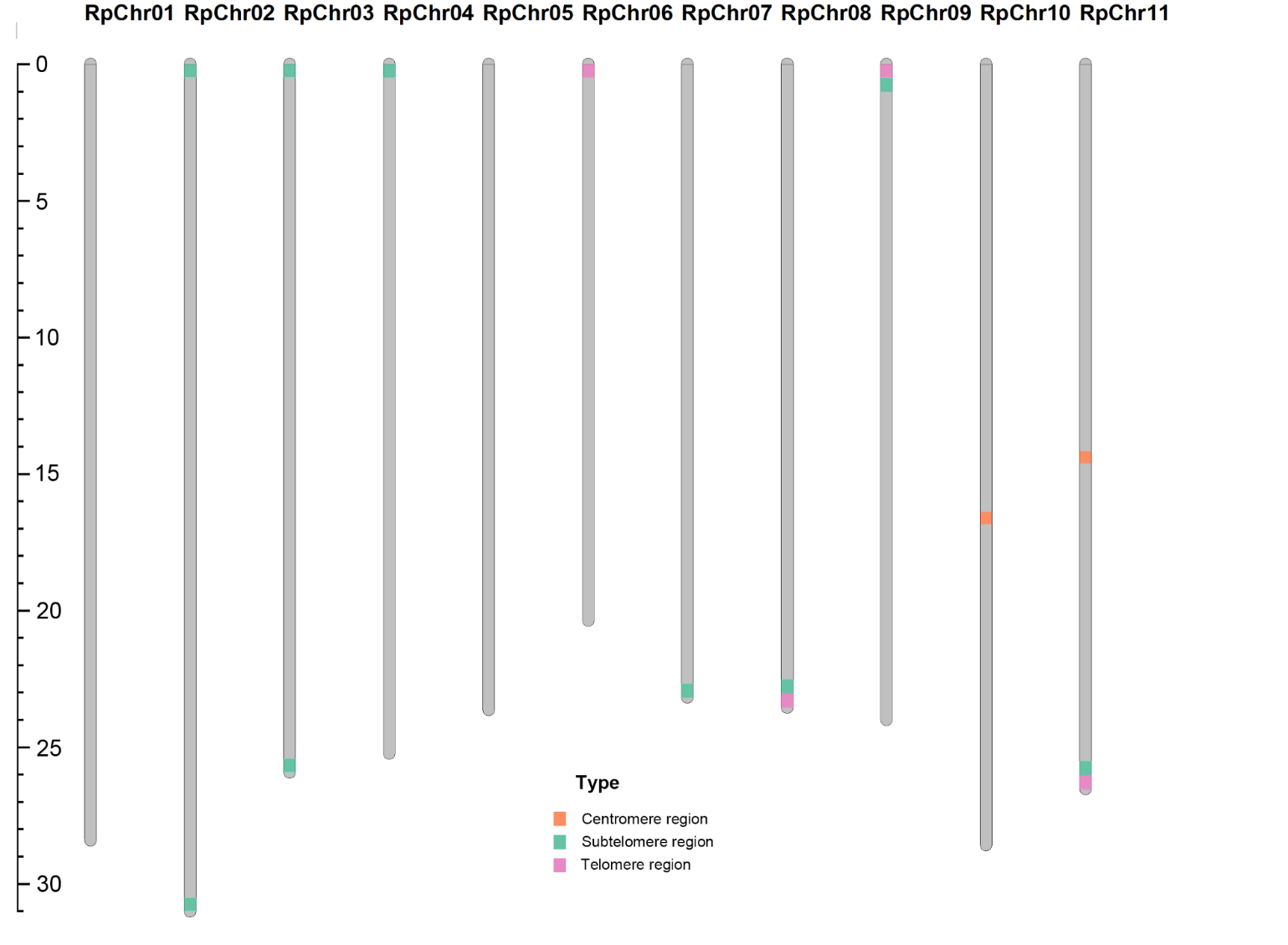


**Figure S6** GO (a) and KEGG (b) enrichment of members of species-specific family in *R. palmatum*
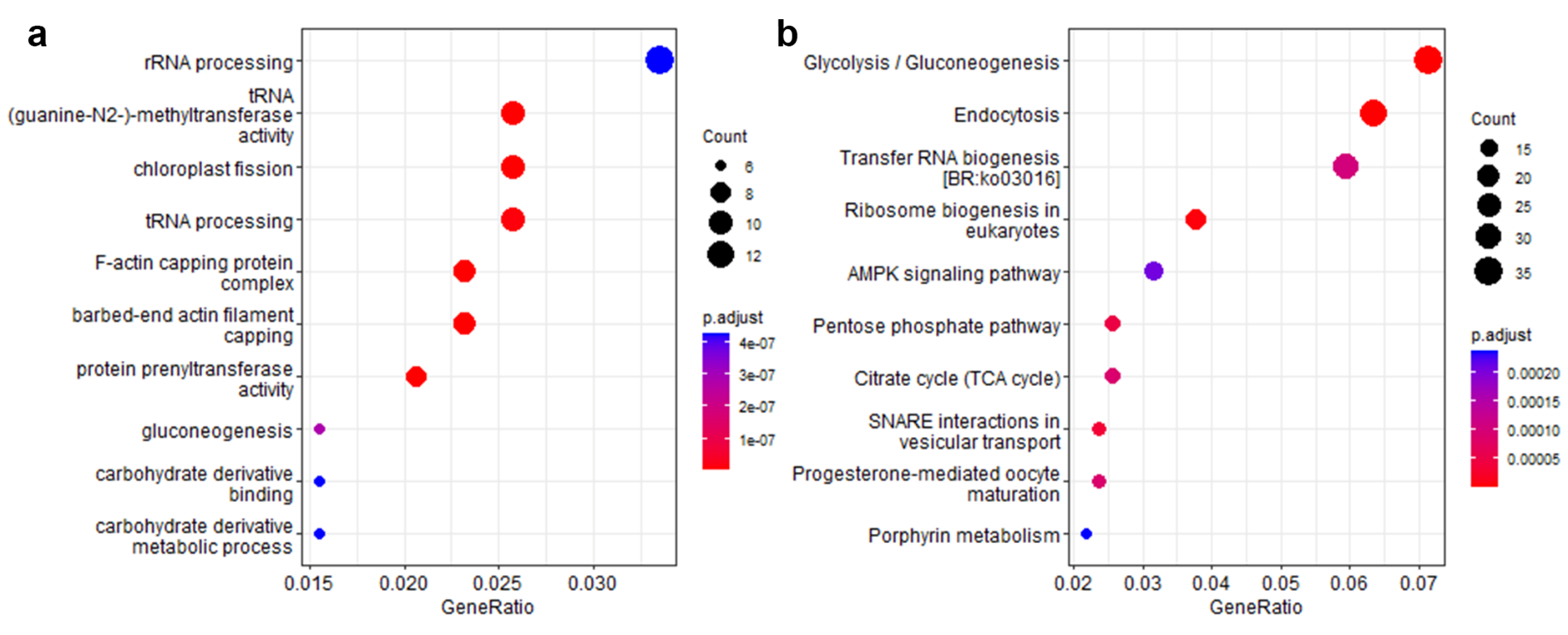


**Figure S7** Phylogenetic relationships inferred from different gene family sets

Bootstrap support (BS) values for each node are shown on the branch of trees in (a) and (b)

Local posterior probability (LPP) values for each node are shown on the branch of trees in (c)-(j)

(a) based on 141 SSC gene families using concatenation ML approaches

(b) based on 344 MSC gene families using concatenation ML approaches

(c)-(f) based on individual gene trees of 1885 LC3 gene families using coalescent phylogenetic approaches

(c) no nodes were collapsed before inputted into coalescent phylogenetic analysis

nodes with bootstrap values (d) less than 10, (e) less than 50, (f) less than 70 were collapsed before inputted into coalescent phylogenetic analysis

(g)-(j) based on individual gene trees of 6218 LC12 gene families using coalescent phylogenetic approaches

(g) no nodes were collapsed before inputted into coalescent phylogenetic analysis

nodes with bootstrap values (h) less than 10, (i) less than 50, (j) less than 70 were collapsed before inputted into coalescent phylogenetic analysis

**
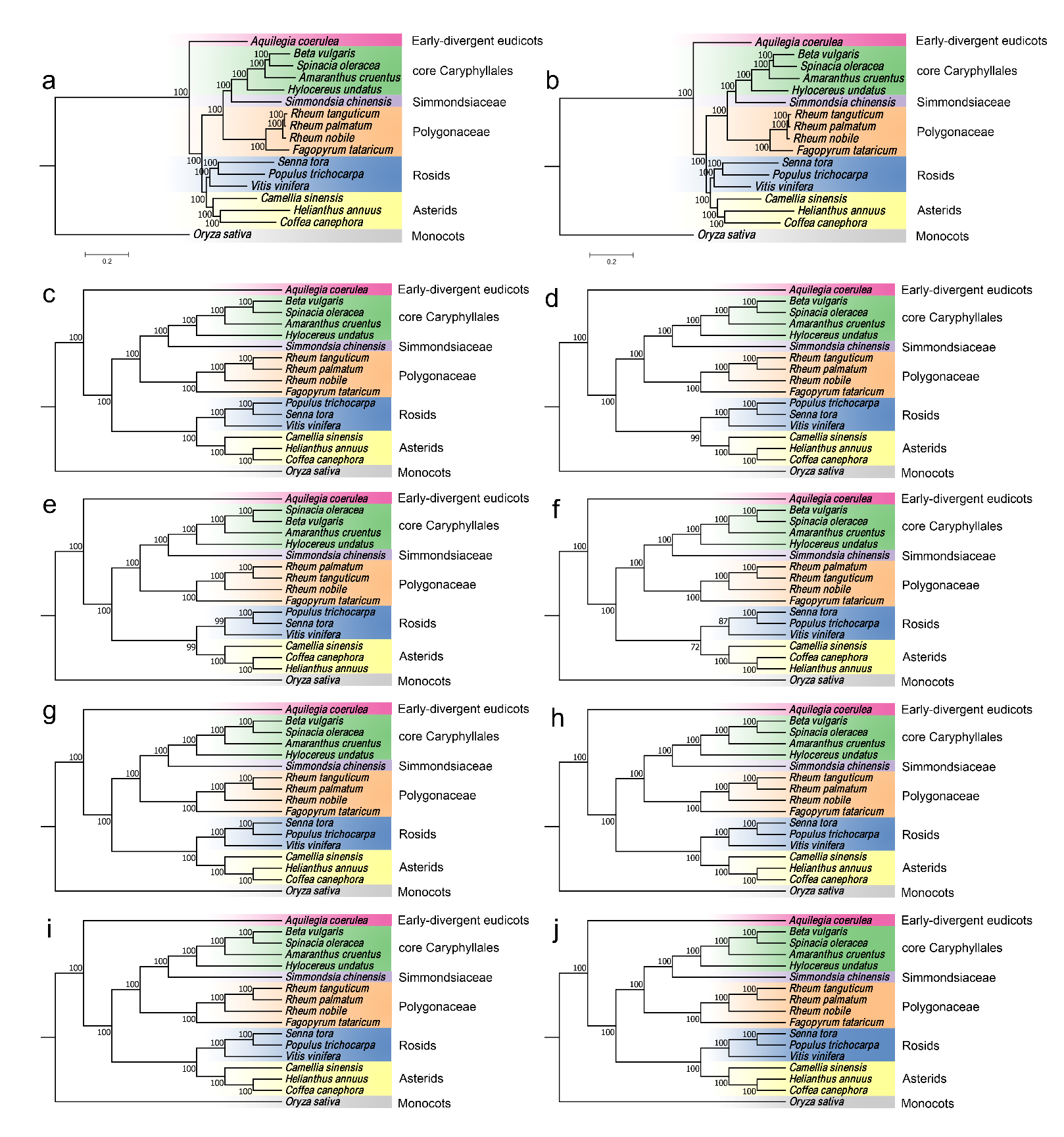
**

**Figure S8** KEGG and GO enrichment of expanded and contracted gene family of RPC

(a) KEGG enrichment of expanded gene family (b) GO enrichment of expanded gene family (c) KEGG enrichment of contracted gene family (b) GO enrichment of contracted gene family


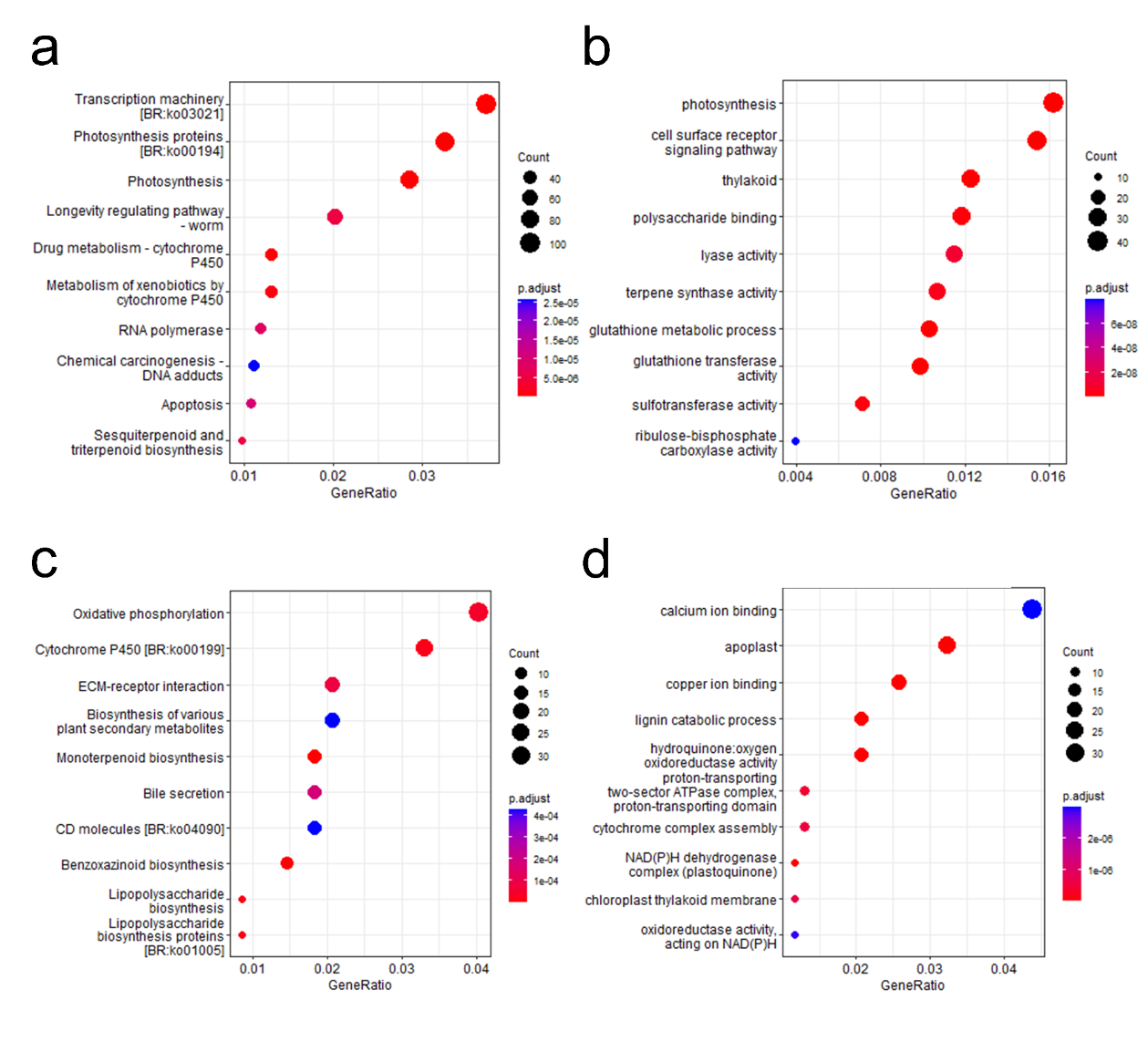


**Figure S9** *K*s distribution of best hit of gene pairs between *Coffea canephora* (Cc), *Haloxylon ammodendron* (Ha), *Simmondsia chinensi*s (Sc), *Vitis vinifera* (Vv), and *R. palmatum* (Rp) and within these species


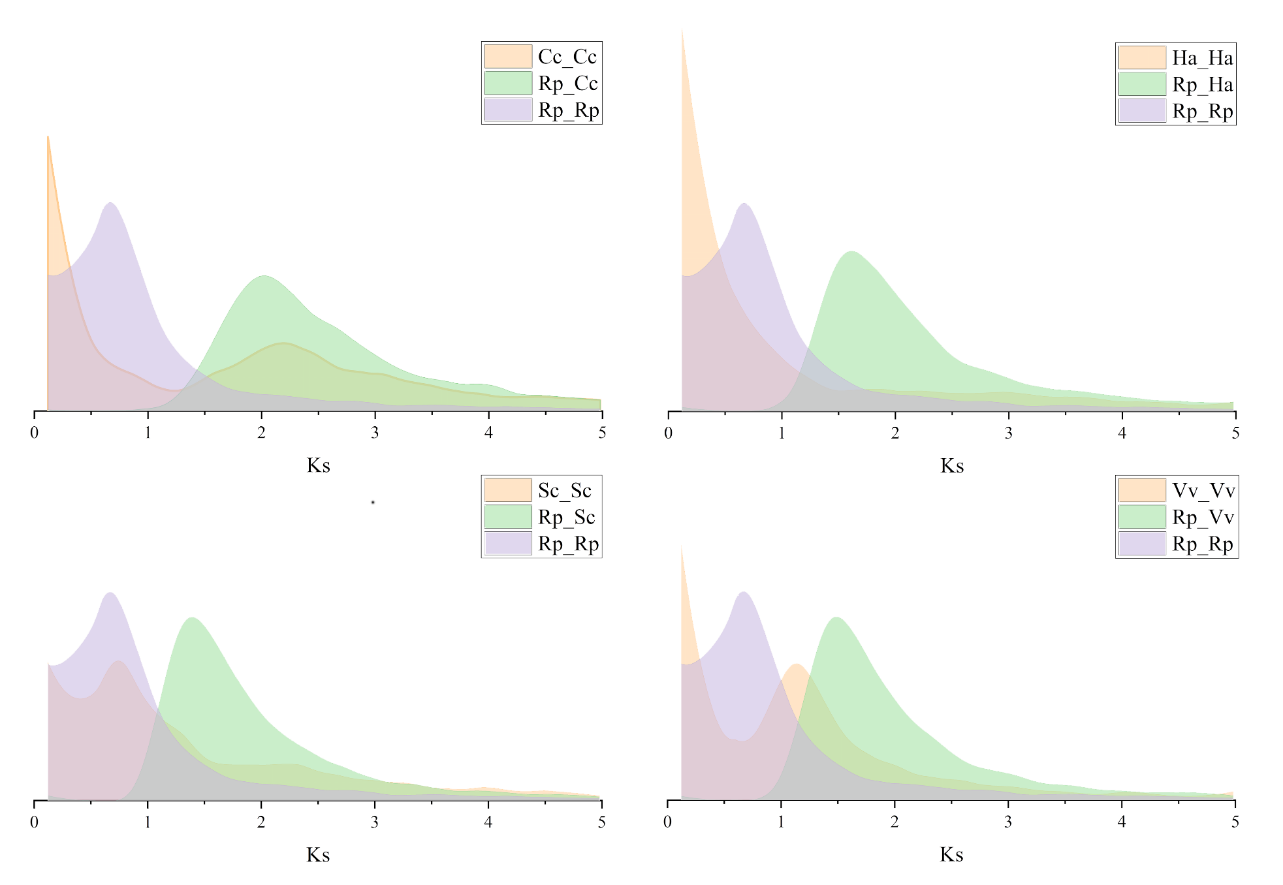


**Figure S10** 4DTv distribution of best hit of gene pairs between and within *Fagopyrum tataricum* (Ft) and *R. palmatum* (Rp)





**Figure S11** 4DTv distribution of best hit of gene pairs between *Coffea canephora* (Cc), *Simmondsia chinensis* (Sc), *Haloxylon ammodendron* (Ha), *Vitis vinifera* (Vv), and *R. palmatum* and within these species


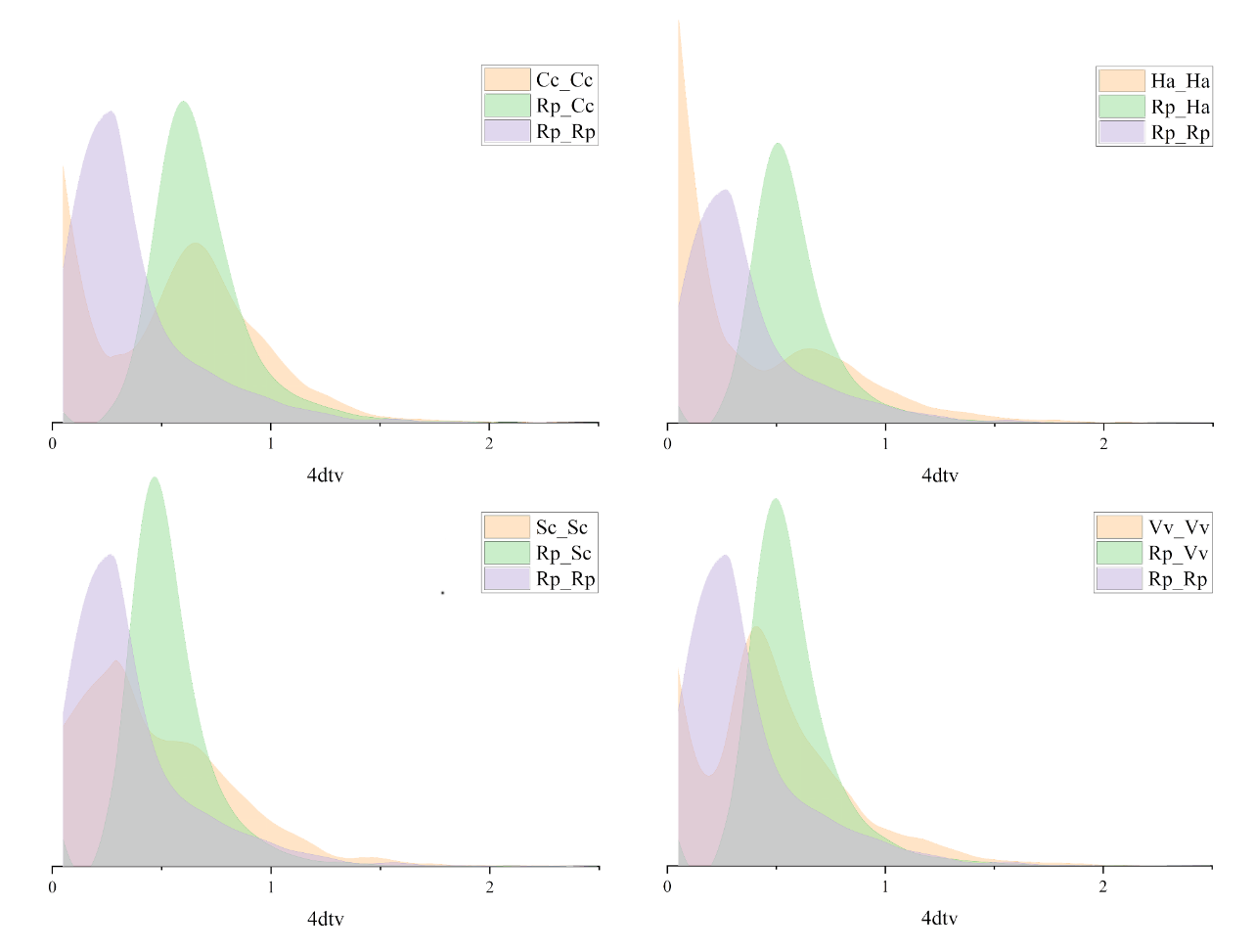


**Figure S12** Collinearity analysis within the *R. palmatum* genome
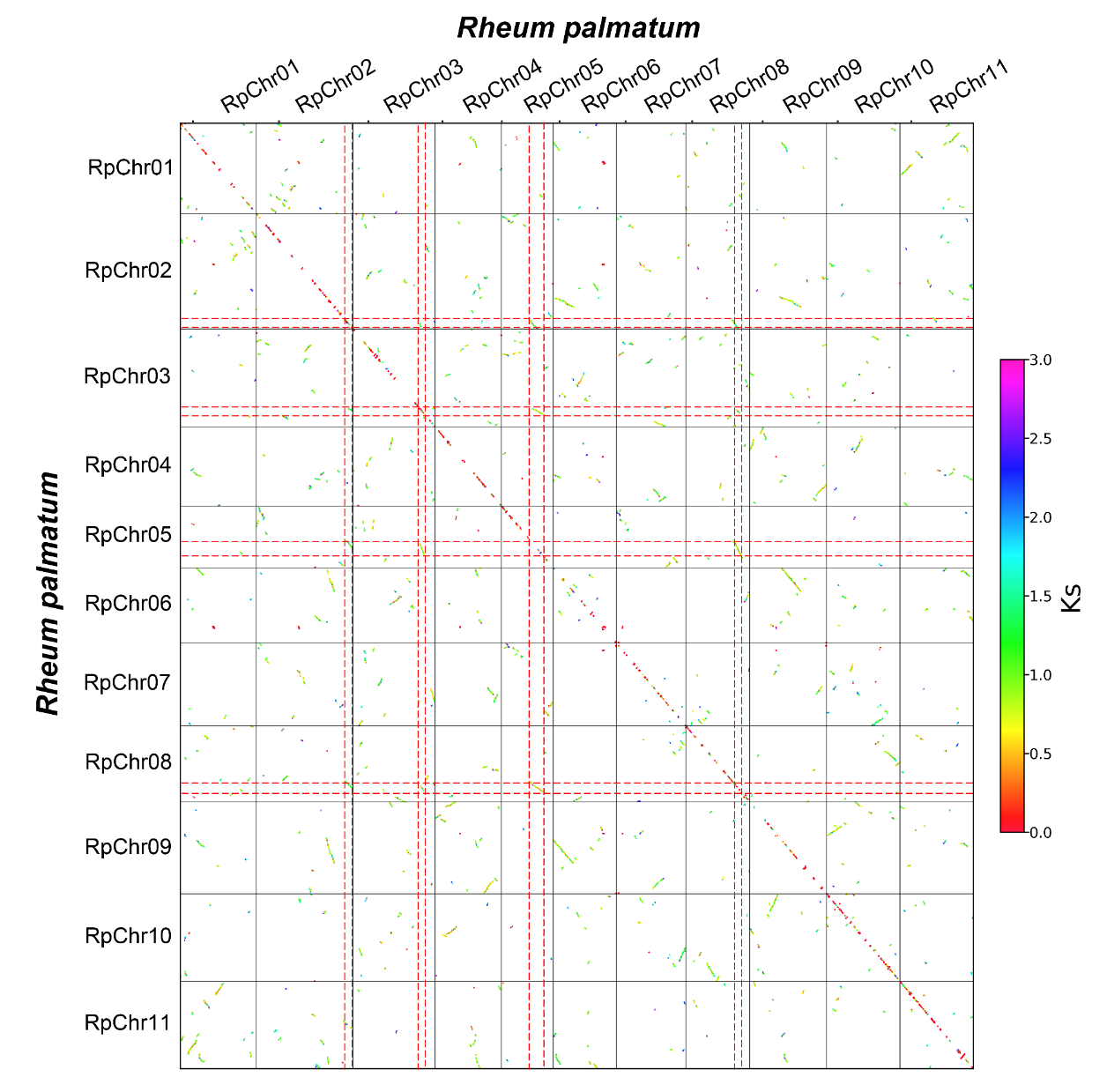


**Figure S13** Collinearity analysis between the genome of *R. palmatum* and *B. vulgaris*


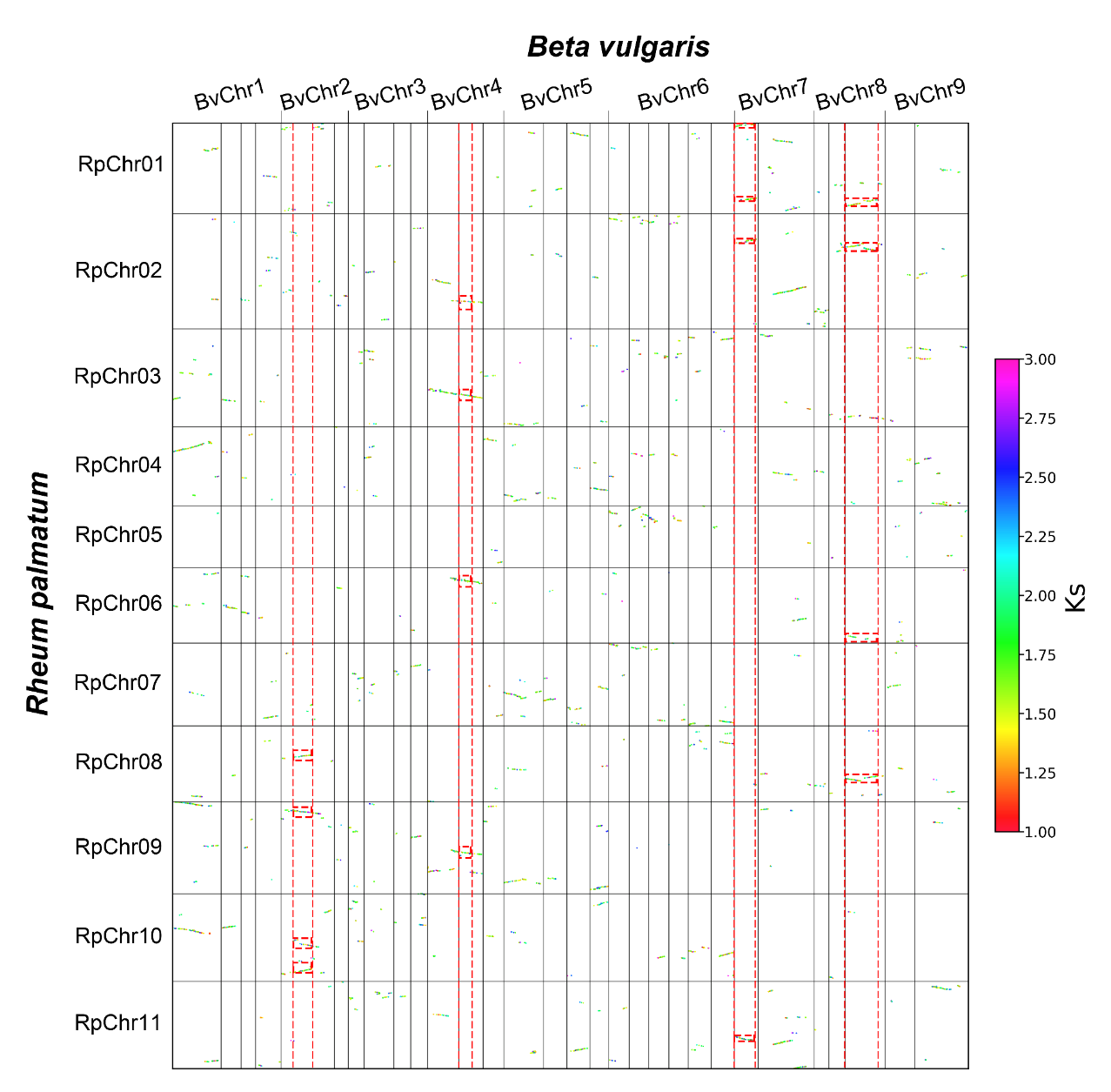


**Figure S14** Collinearity analysis between the genome of *R. palmatum* and *Spinacia oleracea*


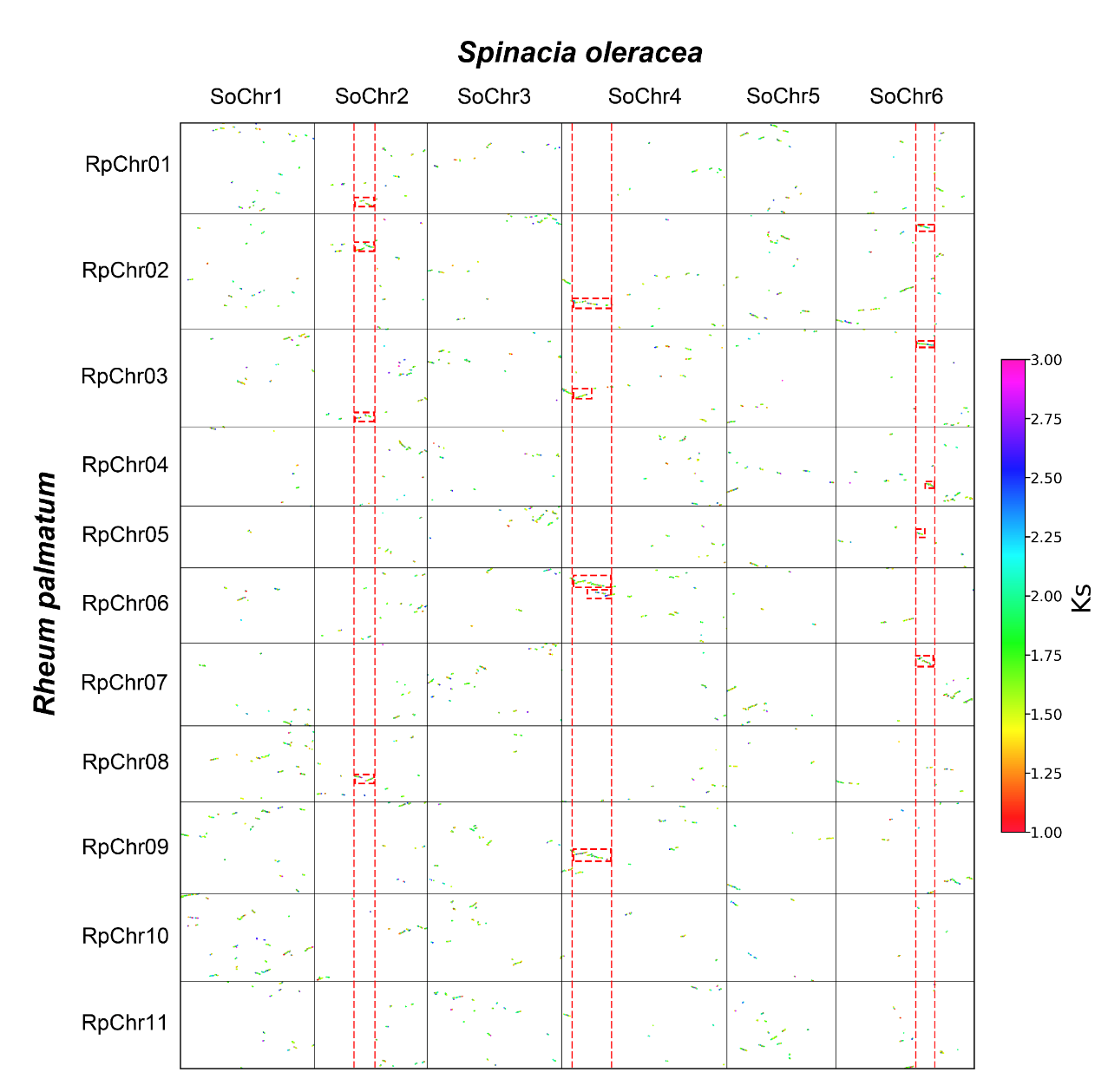


**Figure S15** Collinearity analysis between the genome of *R. palmatum* and *V. vinifera*


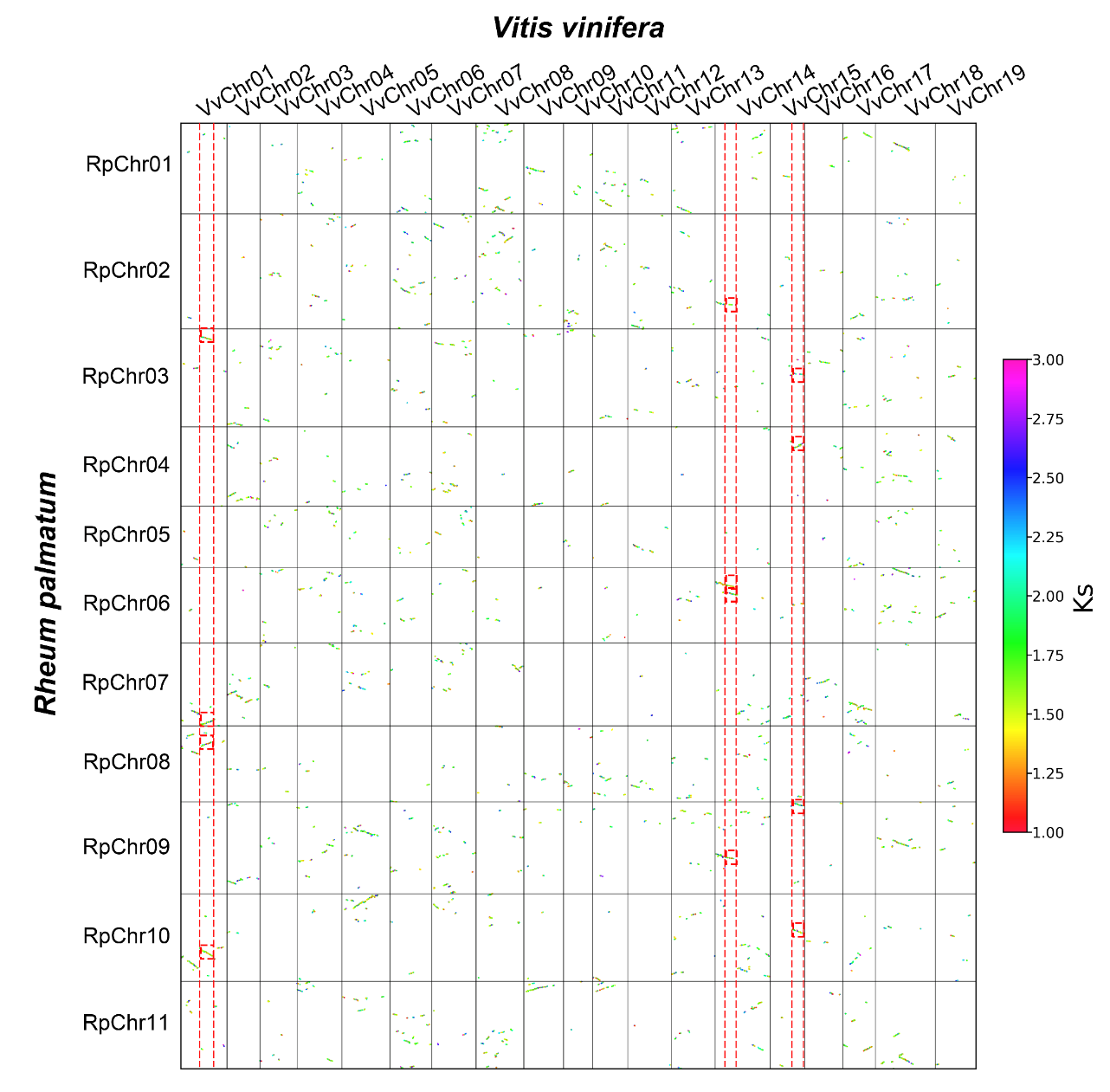


**Figure S16** Collinearity analysis between the genome of *R. palmatum* and *Hylocereus undatus*


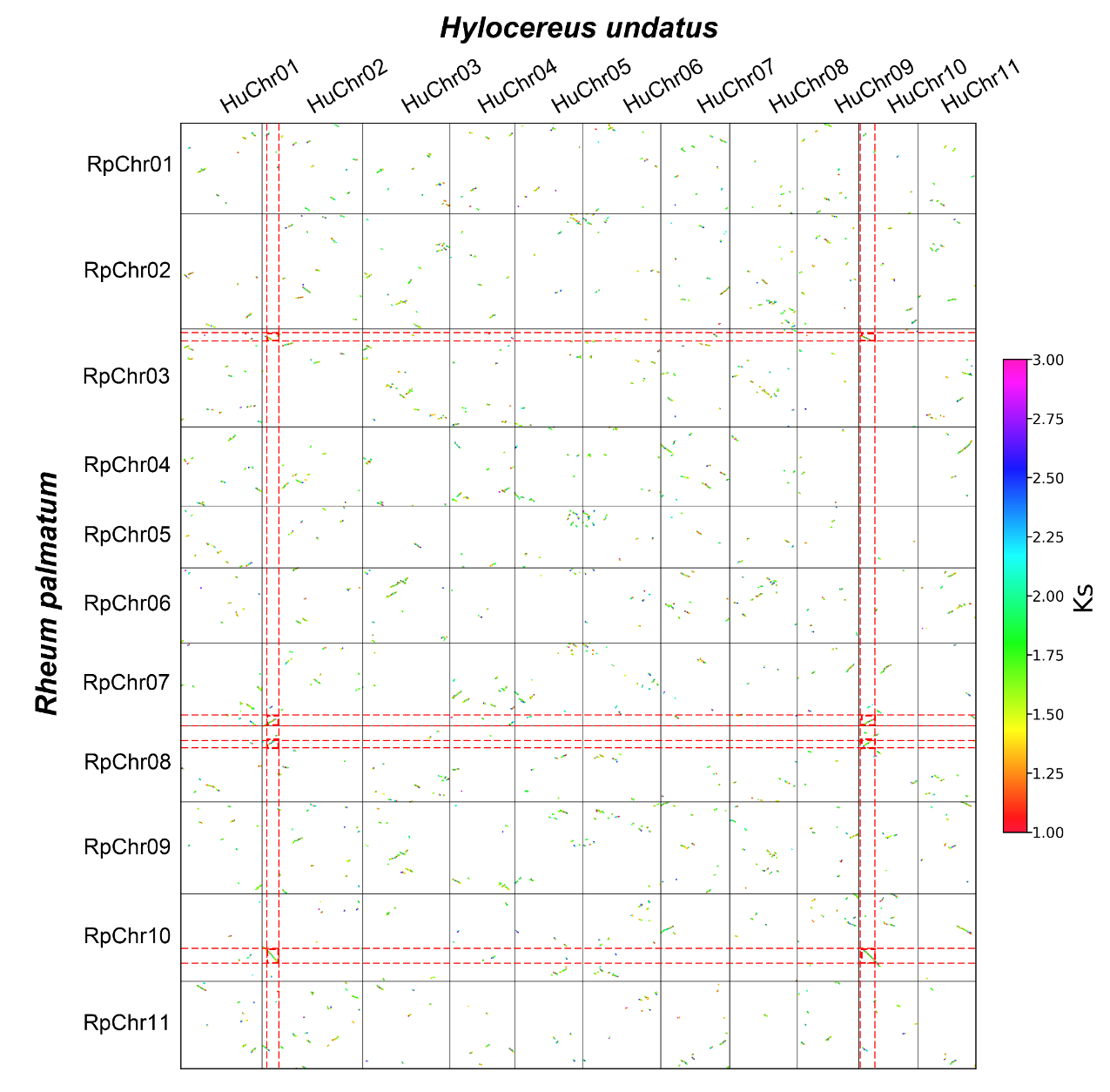


**Figure S17** Collinearity analysis between the genome of *R. palmatum* and *F. tataricum*


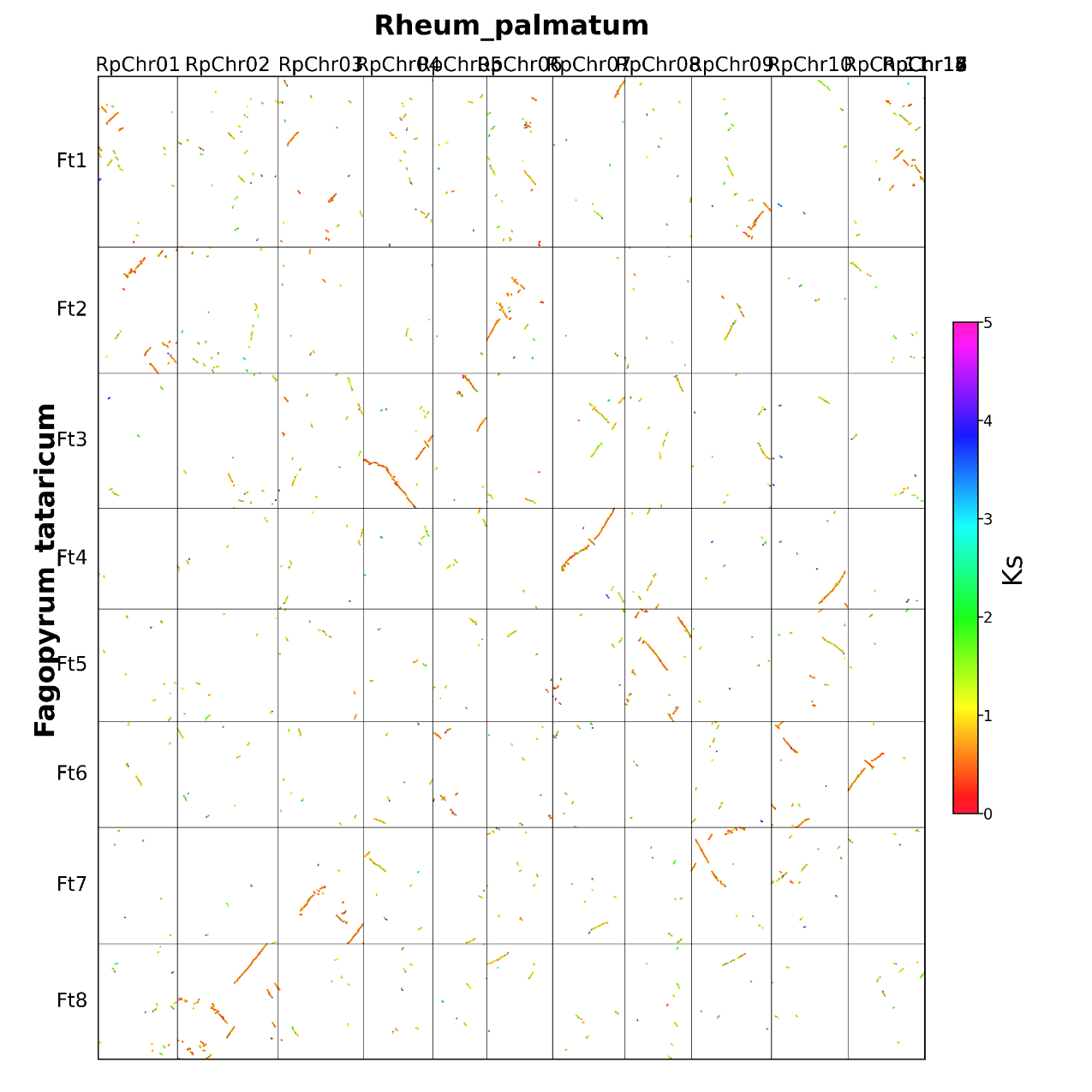


**Figure S18** Collinearity analysis between the genome of *R. palmatum* and *O. digyna*


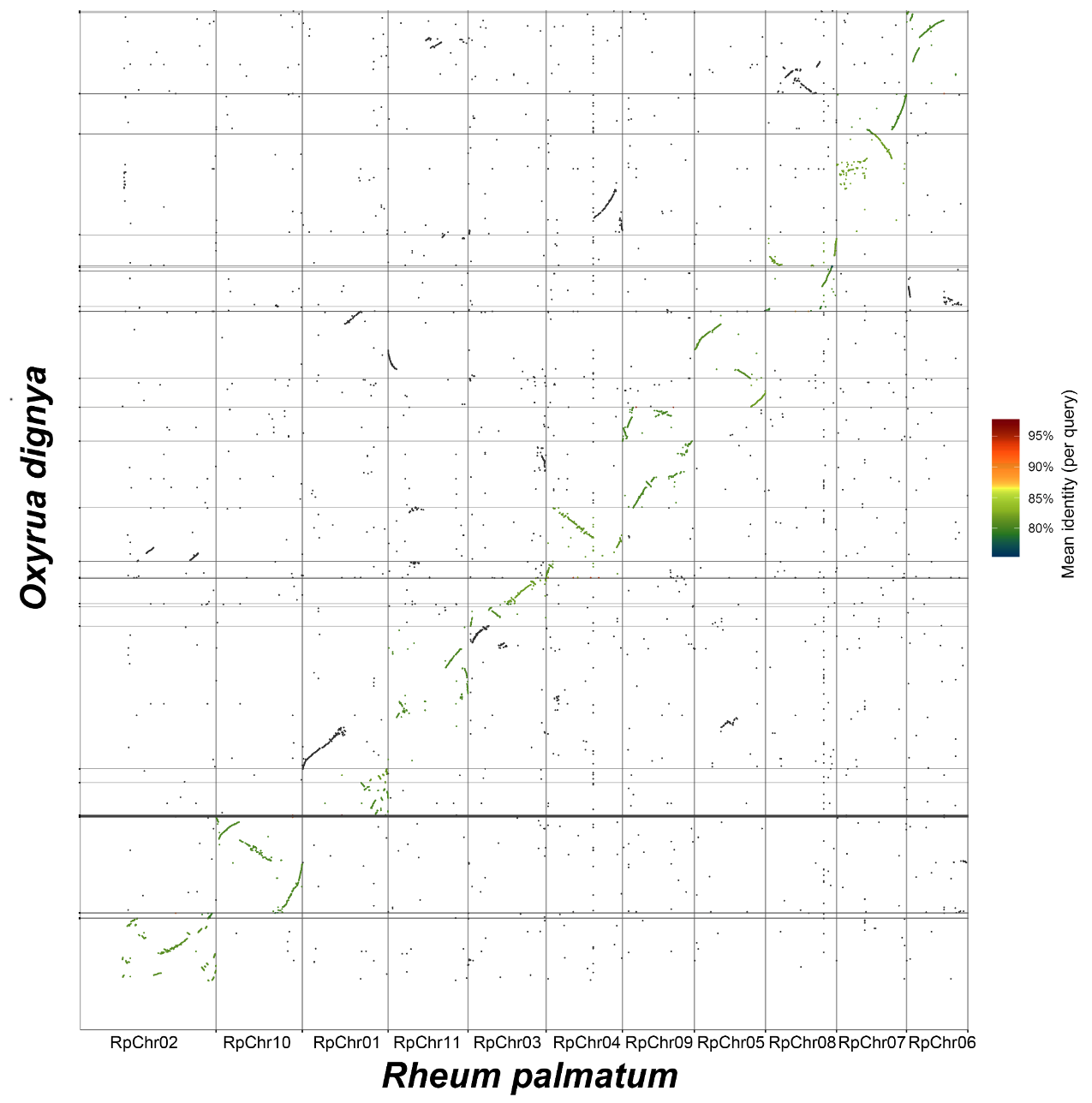


**Figure S19** Collinearity analysis between the genome of *R. palmatum* and *R. nobile* Feng2049


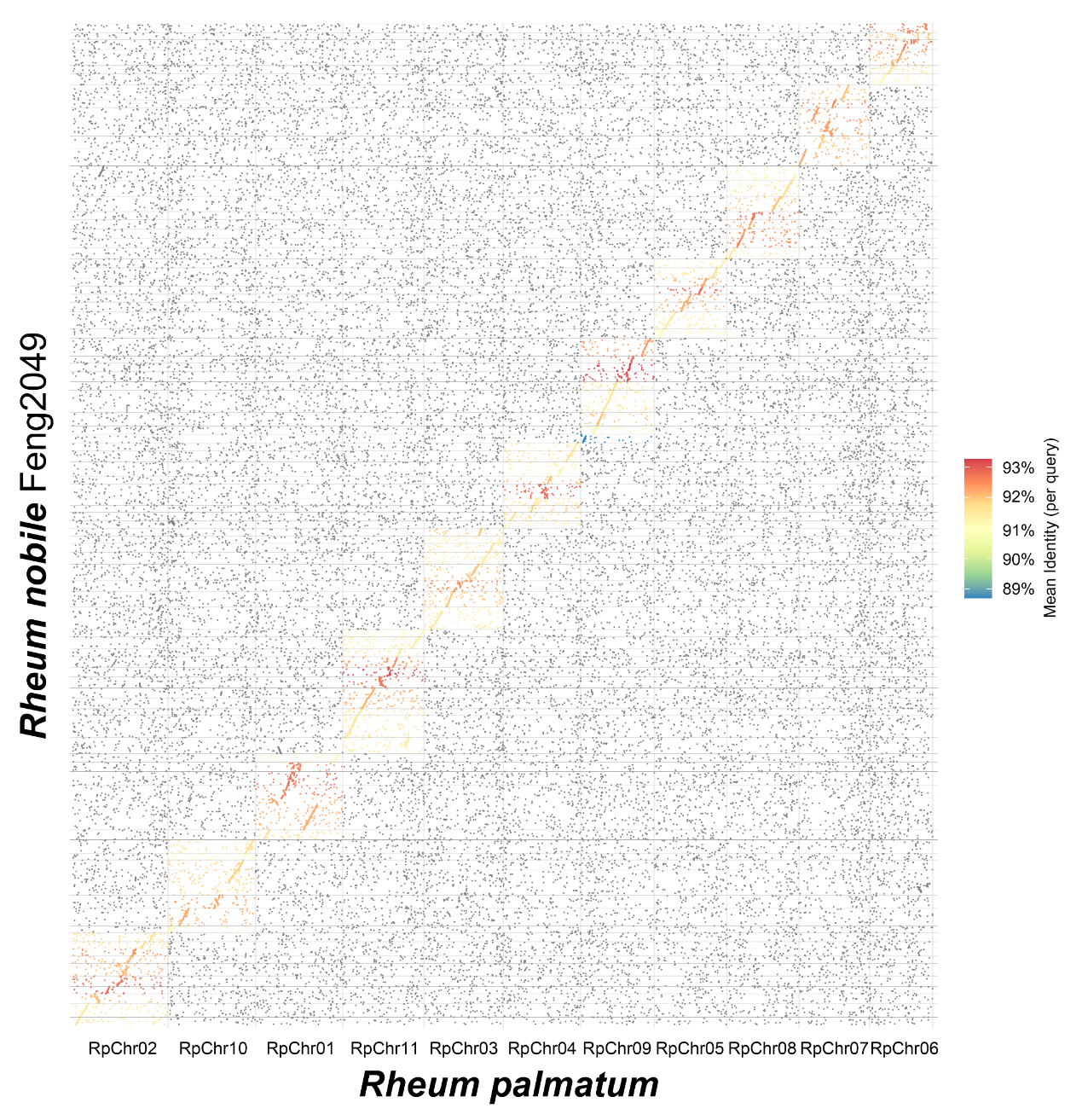


**Figure S20** Anthraquinone and gallic acid biosynthetic pathway and the expression level of related genes in four different organs (R, root. S, stem, L, leaf, F, flower) of *R. palmatum*

PEP: phosphoenol pyruvate; E4P: erythrose 4-phosphate; DAHPS: 3-deoxy-7-phosphoheptulonate synthase; DAHP: 3-deoxy-7-phosphoheptulonate; DHQS: 3-dehydroquinate synthase; DHQ: 3-dehydroquinate; DHD-SHD: 3-dehydroquinate dehydratase--shikimate 5-dehydrogenase; 3DHS: 3-dehydroshikimate; SK: shikimate kinase; SK-3-P: shikimate 3-phosphate; EPSPS: 3-phosphoshikimate 1-carboxyvinyltransferase; EPSP: 5-*O*-(1-carboxyvinyl)-3-phosphoshikimate; CS: chorismate synthase; menF: menaquinone-specific isochorismate synthase; PHYLLO: isochorismate synthase--2-succinyl-5-enolpyruvyl-6-hydroxy-3-cyclohexene-1-carboxylate synthase--2-succinyl-6-hydroxy-2,4-cyclohexadiene-1-carboxylate synthase--*o*-succinylbenzoate synthase; TPP: thiamin diphosphate; OSB: o-succinylbenzoate; AAE14: *o*-succinylbenzoic acid---CoA ligase; menB: naphthoate synthase; menI: 1,4-dihydroxy-2-naphthoyl-CoA hydrolase; DHNA: 1,4-dihydroxy-2-naphthoyl-CoA; G-3-P: *D*-glyceraldehyde 3-phosphate; DXS: 1-deoxy-*D*-xylulose-5-phosphate synthase; DXP: 1-deoxy-*D*-xylulose 5-phosphate; DXR: 1-deoxy-*D*-xylulose-5-phosphate reductoisomerase; MEP: methyl erythritol phosphate; CDP-MES: 2-C-methyl-D-erythritol 4-phosphate cytidylyltransferase; CDP-ME: 4-(cytidine 5'-diphospho)-2-C-methyl-*D*-erythritolt; CDP-MEK: 4-diphosphocytidyl-2-C-methyl-*D*-erythritol kinase; CDP-MEP: 2-phospho-4-(cytidine 5'-diphospho)-2-C-methyl-*D*-erythritol; ME-cPPs: 2-C-methyl-*D*-erythritol 2,4-cyclodiphosphate synthase; ME-cPP: 2-C-methyl-*D*-erythritol 2,4-cyclodiphosphate; HMB-PPS: (E)-4-hydroxy-3-methylbut-2-enyl-diphosphate synthase; HMB-PP: (E)-4-hydroxy-3-methylbut-2-enyl-diphosphate; ACAT: acetyl-CoA C-acetyltransferase; HMGS: hydroxymethylglutaryl-CoA synthase; HMG-CoA: (S)-3-hydroxy-3-methylglutaryl-CoA; HMGR: 3-hydroxy-3-methylglutaryl-CoA reductase; MVK: mevalonate kinase; PMEV: (R)-5-phosphomevalonate; MVAK2: phosphomevalonate kinase; PPMEV: (R)-5-diphosphomevalonate; MVD: diphosphomevalonate decarboxylase; DMAPP: dimethylallyl diphosphate; IPPI: isopentenyl diphosphate delta-isomerase; IPP: isopentenyl diphosphate; ispS: isoprene synthase; ACC: acetyl-CoA carboxylase


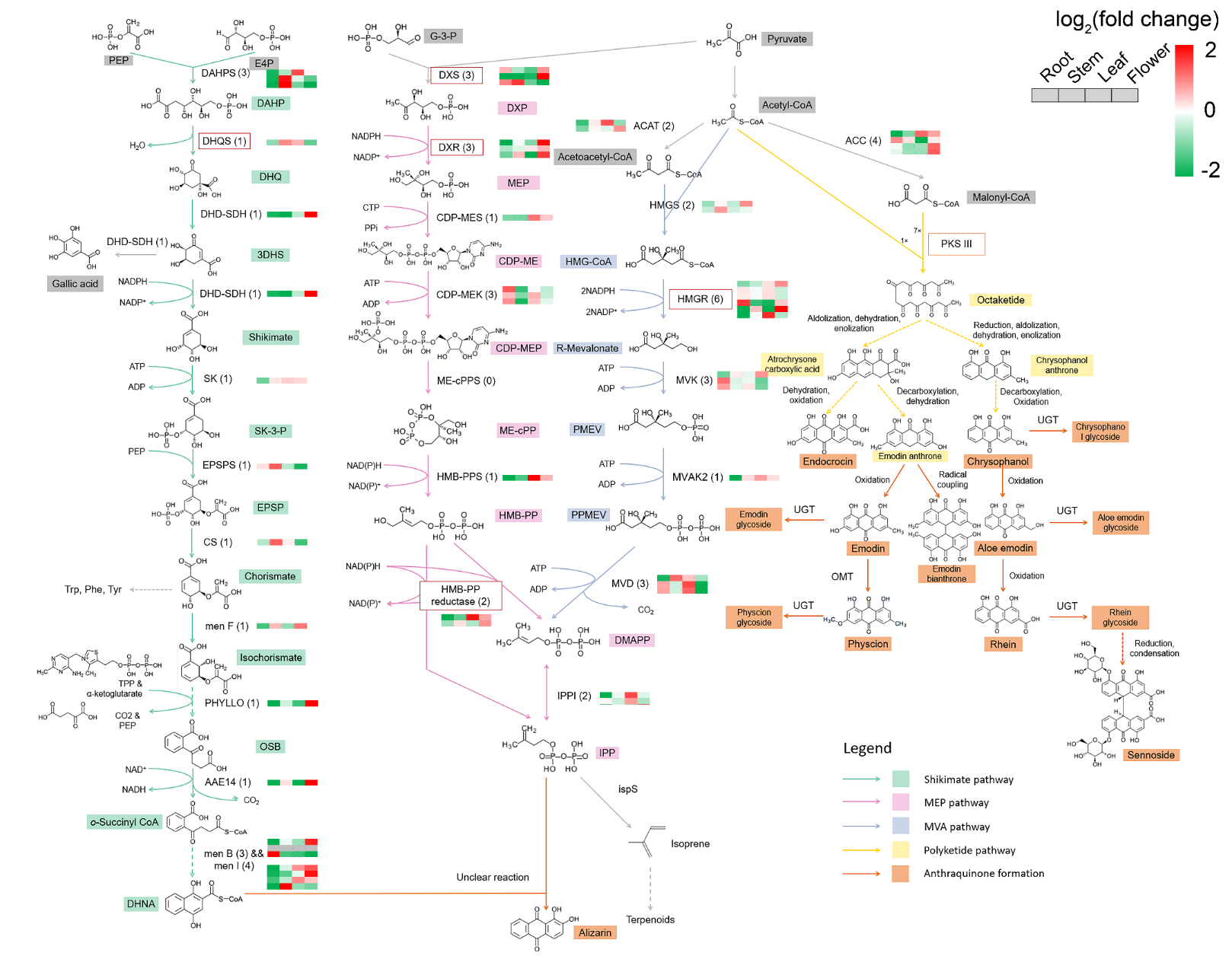


**Figure S21** Catechin, stilbenes and flavonoids biosynthetic pathway and the expression level of related genes in four different organs (R, root. S, stem, L, leaf, F, flower) of *R. palmatum*

PAL: phenylalanine ammonia-lyase; 4CL: 4-coumarate--CoA ligase; 4CH: trans-cinnamate 4-monooxygenase; STS: stilbene synthase; CHS: chalcone synthase; CHI: chalcone isomerase; FNS I: flavone synthase I; F3H: flavanone 3-dioxygenase; FLS: flavonol synthase; HCDBR: hydroxycinnamoyl-CoA reductase; PGT: phlorizin synthase; CHR: 6'-deoxychalcone synthase; F3’H: flavonoid 3'-monooxygenase; HIDH: 2-hydroxyisoflavanone dehydratase; HI4OMT: isoflavone 4'-O-methyltransferase; IFR: 2'-hydroxyisoflavone reductase; VR: vestitone reductase; PTS: pterocarpan synthase; PTR: pterocarpan reductase; FL7GT: ; F3’5’H: flavonoid 3',5'-hydroxylase; DFR: dihydroflavonol-4-reductase; ANS: anthocyanidin synthase; LAR: leucoanthocyanidin reductase; ANR: anthocyanidin reductase; AOMT: flavonoid *O*-methyltransferase


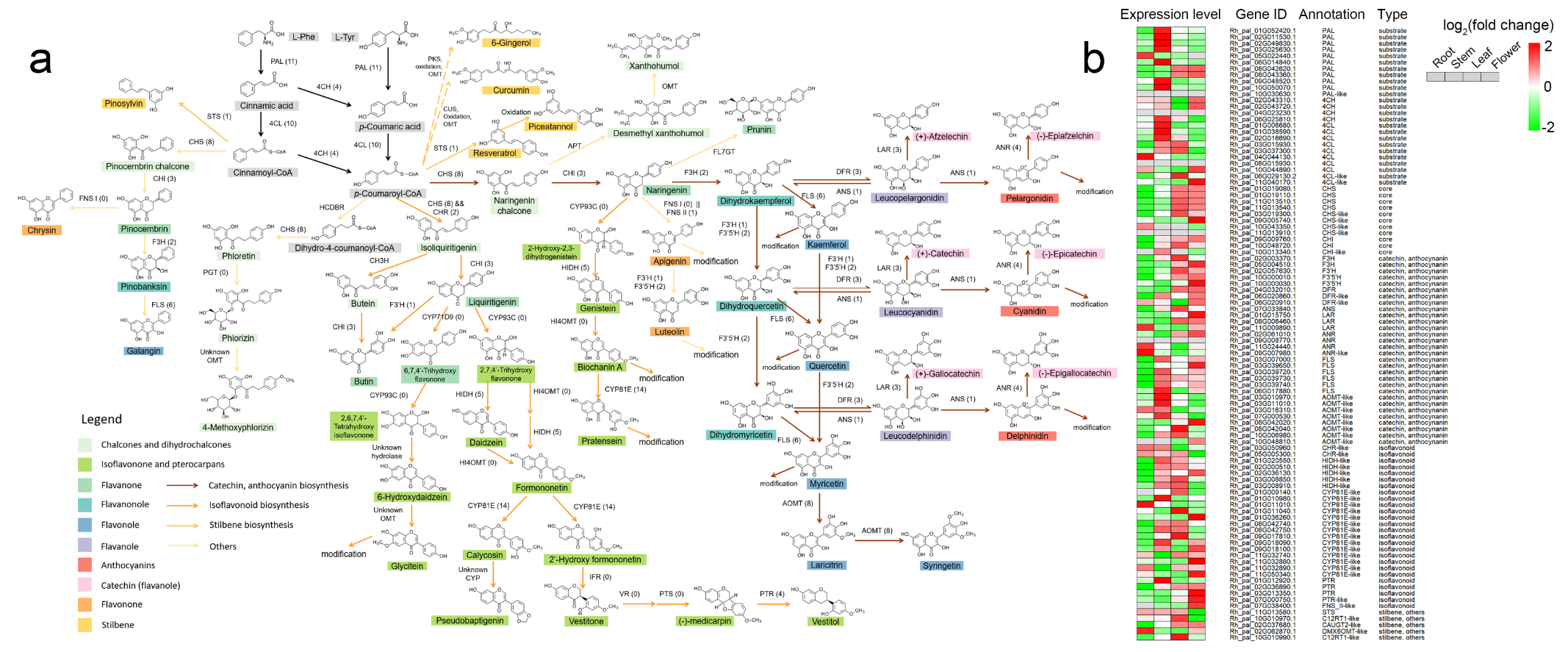


**Figure S22** Phylogeny of *CYP* gene family of *R. palmatum*


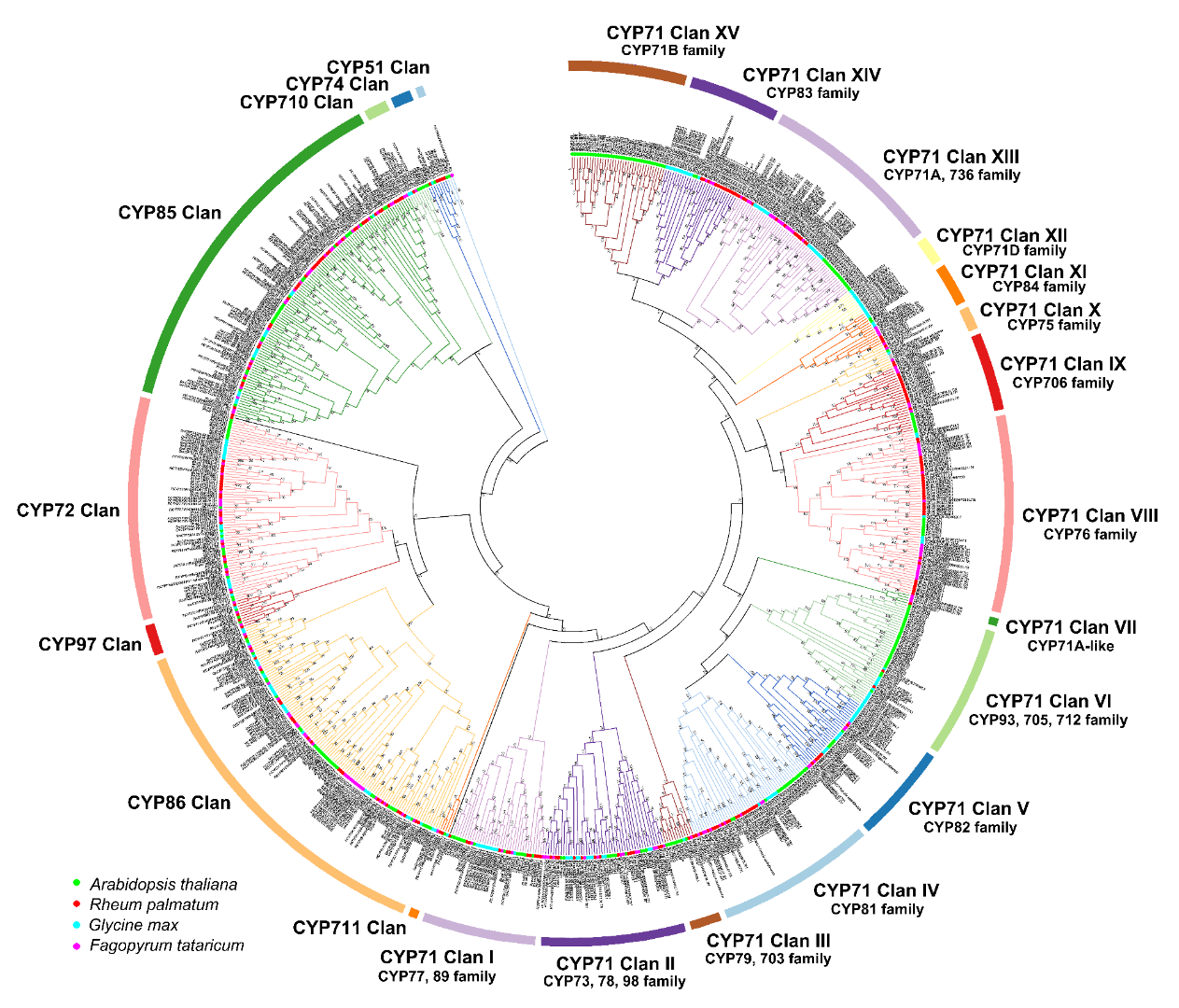


**Figure S23** Phylogeny of *UGT* gene family of *R. palmatum*


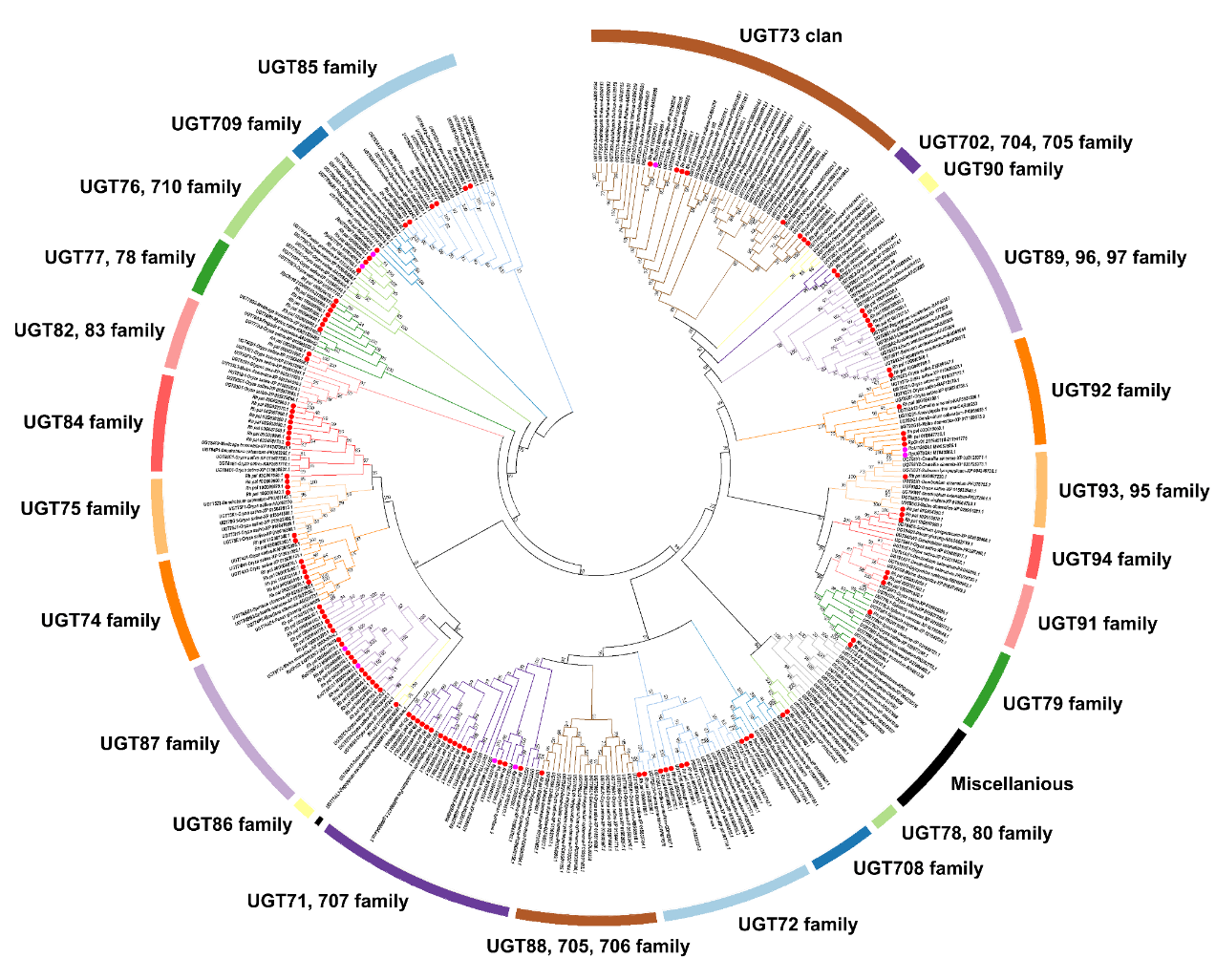


**Figure S24** Phylogeny of *OMT* gene family of *R. palmatum*


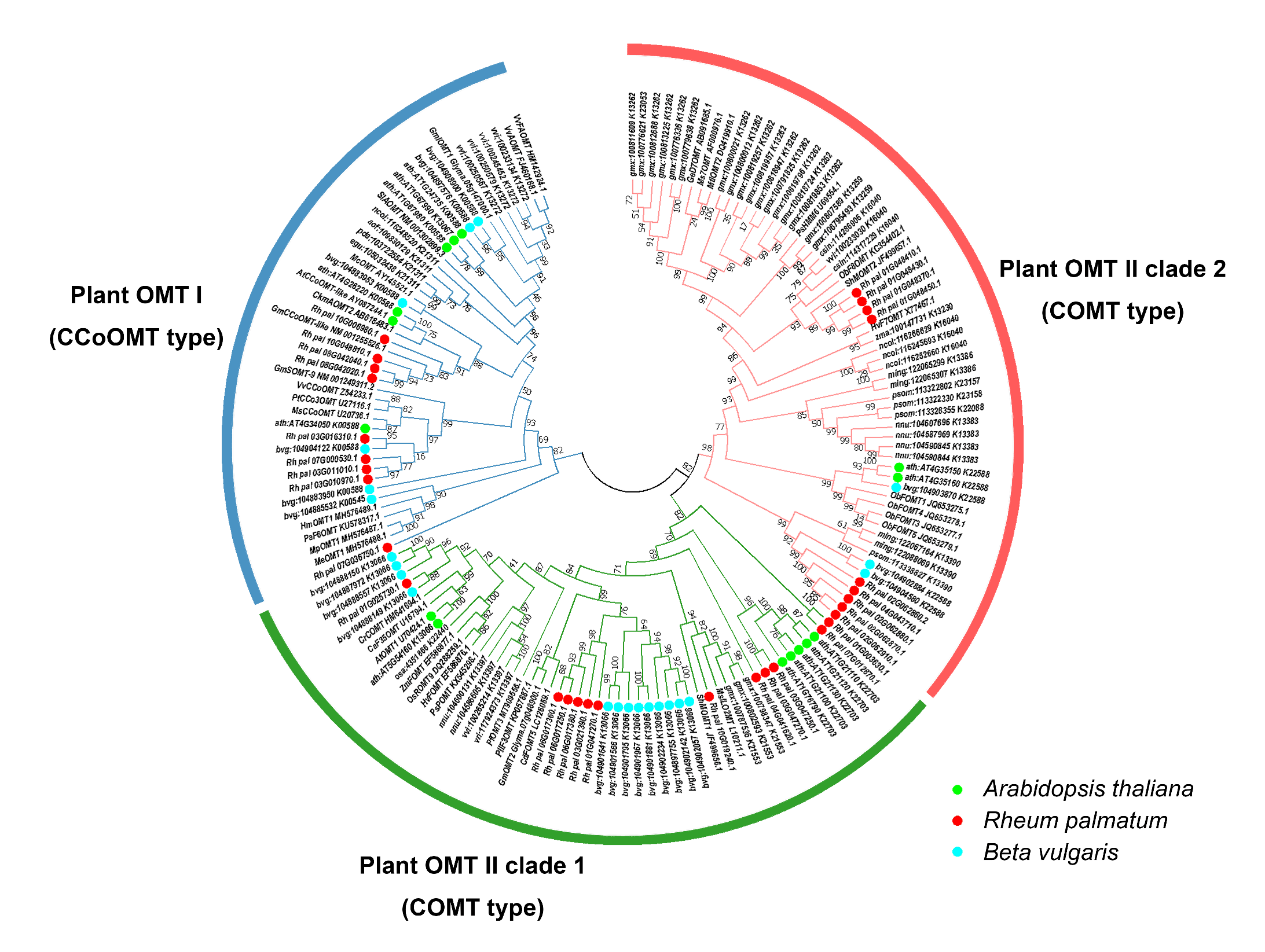


**Figure S25** PKS III family members of *R. palmatum* (a) The distribution on pseudochromosome. Tandem duplication gene pairs were shown with red backgrounds. (b) The expression pattern in four different organs (c) The gene structure of *PKS* genes (d) Comparison of the number of PKS members among representative Caryophyllales species


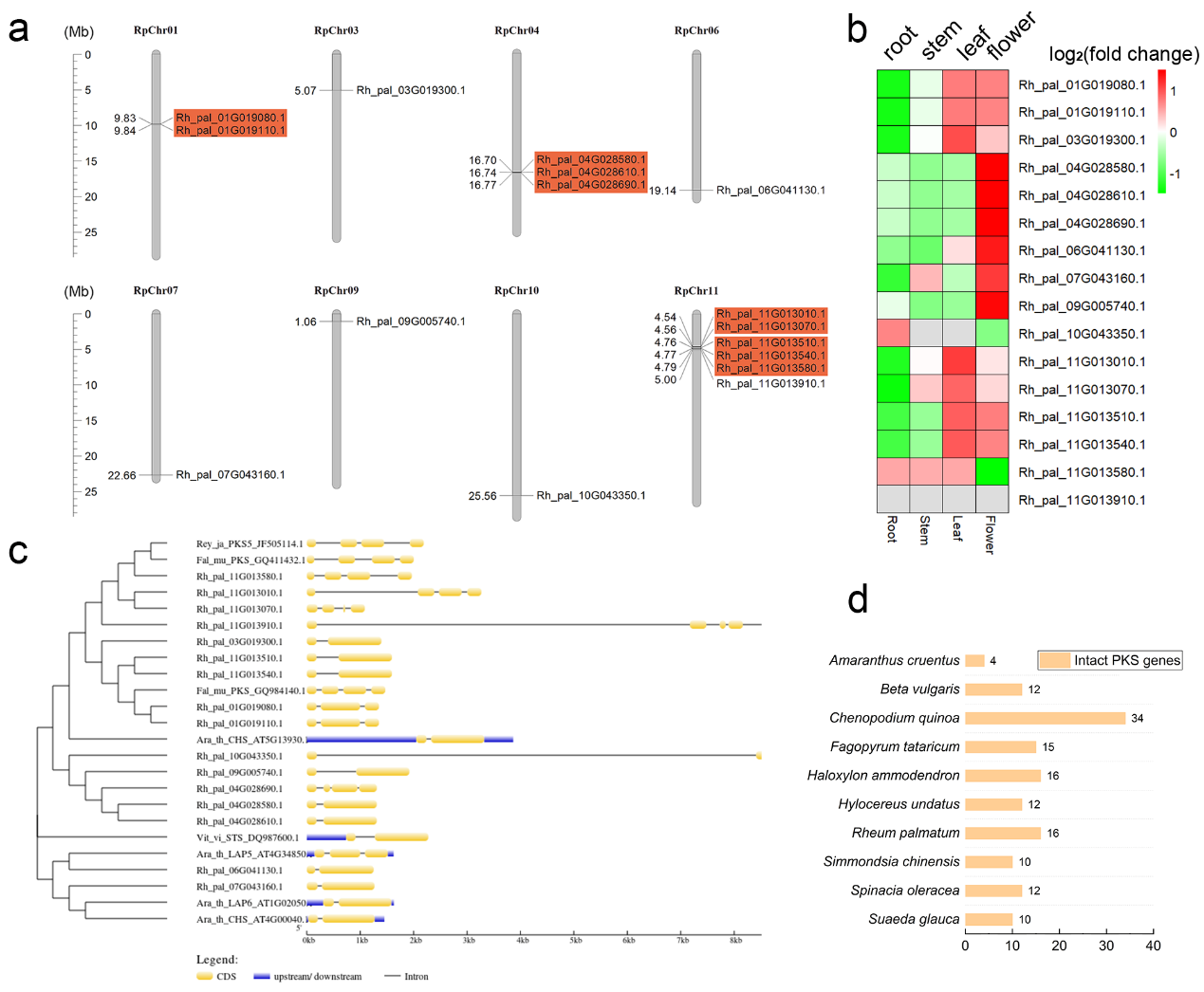


**Figure S26** Comparison of the key residues between RpALS and other OKSs


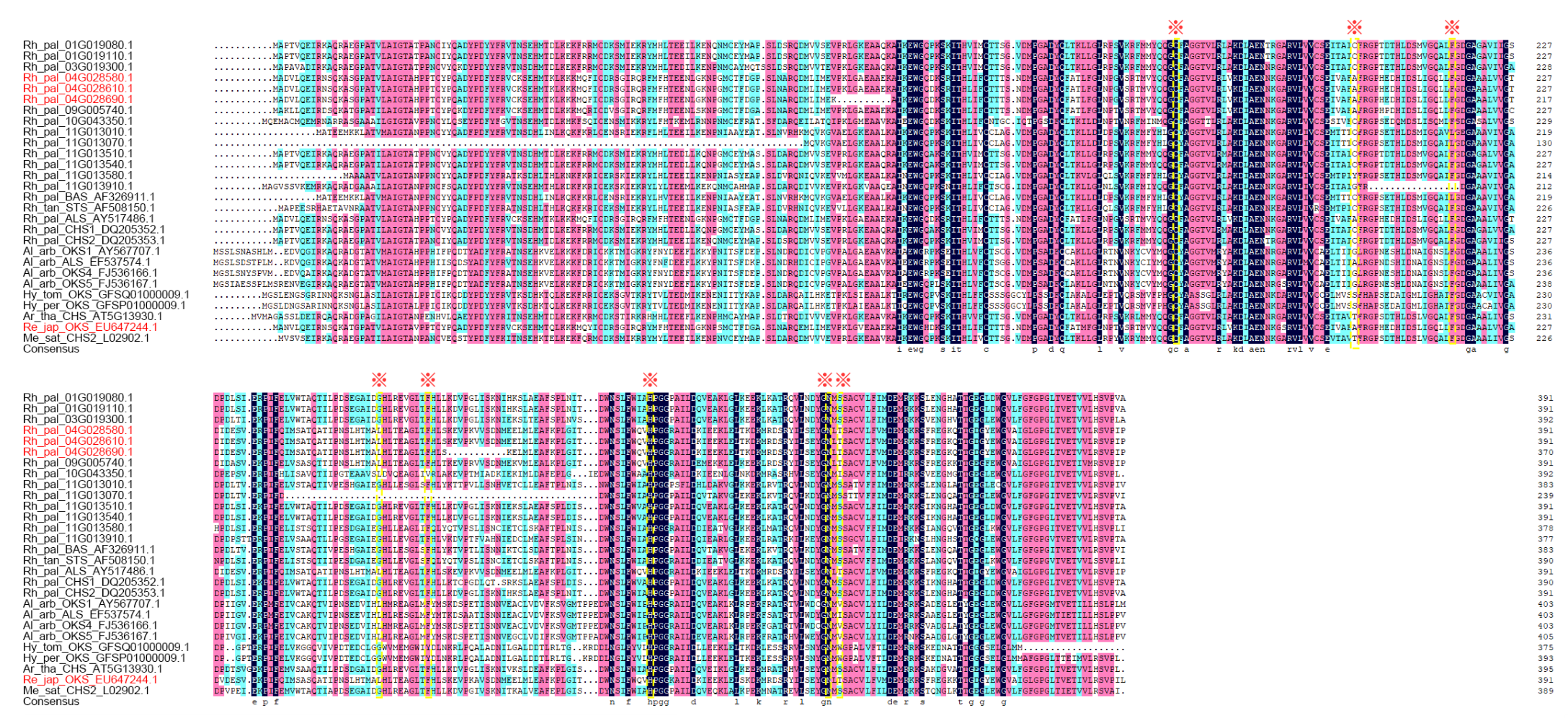


**Figure S27** Genome size and GC% of 1,107 plant genomes retrieved from NCBI database
